# Background selection in recombining genomes and its consequences for the maintenance of variation in complex traits

**DOI:** 10.1073/pnas.2513613123

**Published:** 2026-03-31

**Authors:** Xinyi Li, Jeremy J. Berg

**Affiliations:** ^a^Committee on Genetics, Genomics, Systems and Biology, University of Chicago, Chicago, IL 60637; ^b^Department of Human Genetics, University of Chicago, Chicago, IL 60637

**Keywords:** population genetics, background selection, mutation–selection–drift balance

## Abstract

Background selection (BGS)—the reduction of linked neutral diversity via the purging of deleterious mutations—is a pervasive force in genomic evolution. However, its impact on complex phenotypes remains poorly understood because classical theory treats fitness effects as fixed rather than emerging from a phenotype-to-fitness map. Here, we investigate the impact of BGS across three phenotypic selection frameworks: exponential directional, a liability threshold model, and stabilizing selection. First, we develop an effectively nonrecombining block approximation for the site frequency spectrum (SFS) and show that this framework accurately describes the skew in the SFS in the weak mutation regime typical of humans. Second, we show that phenotypic impacts of BGS depend on how selection is coupled across loci. In the liability threshold model, strong synergistic epistasis generates a global compensation mechanism—driven by tiny shifts in the mean phenotype—that propagates BGS effects to strongly selected variants otherwise immune to linked selection. This coupling reduces genetic variance across almost the entire effect-size distribution by an amount determined by a nonlinear average of local effective population size reductions across the genome. Conversely, under stabilizing selection, BGS can counterintuitively increase genetic variance. This occurs because BGS shifts strongly selected sites into the weakly selected underdominant regime where they persist at intermediate frequencies longer than in the equivalent directional selection model. Our results inform both longstanding evolutionary conversations regarding synergistic epistasis and efforts to model the impact of background selection on individual variants in recombining genomes.

## Significance

Natural selection against deleterious mutations reshapes genetic variation through background selection, a process typically modeled as a local reduction in effective population size. We show that the consequences of this reduction for genetic architecture are mediated by the relationship between phenotype and fitness. Extending background selection theory to complex traits, we show that in models with strong synergistic epistasis, a global compensation mechanism propagates the effects of selection to strongly selected variants. Furthermore, we find that background selection can counterintuitively increase genetic variation under stabilizing selection. Our work shows that the total impact of background selection on functional variation thus depends on the answers to longstanding questions about the ultimate sources of variance in fitness.

Natural selection acting on deleterious mutations reduces genetic variation at linked neutral sites, a process known as background selection (BGS; 1). In the decades since its initial description, BGS has become a standard component of population genetic null models. To a first approximation, BGS acts as a local reduction in the effective population size (B=NeN), which reduces the number of segregating sites and increases the rate of genetic drift (2–4). The magnitude of this reduction depends largely on the local density of functional sites relative to the recombination rate and varies substantially across species. For example, in humans, BGS effects are generally modest [B≈0.6 in the most affected regions, with B≈0.83 on average; (5, 6)], whereas in the more compact *Drosophila* genome, the average reduction is much stronger [mean B≈0.3; (7, 8)]. The existence of broad-scale correlations between recombination rate and diversity across taxa suggests that these linked selection effects are a pervasive feature of genomic evolution across the tree of life (9–11).

However, the standard “reduced local $N_{e}$” approximation provides an incomplete picture of BGS on two key fronts. One is that it fails to capture the distortion of the site frequency spectrum (12). This distortion arises because BGS does not act instantaneously; alleles must persist long enough to “feel” the purging of linked deleterious backgrounds, and the resulting delay creates a skew in the SFS that confounds inferences about both demography and selection (13). Prior work has provided an analytical description of this skew for nonrecombining regions in the strong mutation regime (14) and (15) characterized the distortions to gene genealogies caused by BGS in recombining genomes, but a direct description of the frequency spectrum skew in the recombining case has remained elusive.

The other is that our understanding of how BGS impacts complex phenotypes remains limited, even as empirical studies suggest it shapes genetic architecture. For example, ref. 16 observed that *Caenorhabditis elegans* loci in gene-poor, high-recombination regions explain significantly more gene expression variance. Similarly, in human complex traits, per-SNP heritability appears enriched in regions of strong BGS (17, 18). However, theoretical work has largely been restricted to single-site models in which the fitness effects of individual loci are fixed (19, 20). If the fitness effects of variants are largely mediated by their contributions to polygenic traits, their fitness effects are not fixed, but emerge from the distribution of phenotypes in the population and the relationship between phenotype and fitness (21, 22). Consequently, determining the full impact of BGS requires understanding how local linkage effects interact with the relationship between the phenotype and fitness.

Here, we address both of these limitations. We first develop an effectively nonrecombining block approximation for the dynamics of BGS in recombining genomes, and use it to describe the skew in the frequency spectrum in the weak mutation limit, showing that it is valid broadly in the parameter regime relevant to humans. We then study the impact of BGS in three models of phenotypic mutation–selection–drift balance. We first contrast two models of directional selection: an exponential fitness model, where loci evolve independently, and a liability threshold model, where fitness effects are tightly coupled across loci. We show that in the threshold model, the system responds to BGS through a global compensation mechanism driven largely by the genomic regions experiencing the strongest background selection. Unlike simple local $N_{e}$ rescaling—which impacts only weakly selected sites—this global mechanism propagates the effects of BGS to strongly selected variants that would otherwise be immune to linked selection. Finally, we consider a model of stabilizing selection, the ubiquity of which is motivated by both direct (23, 24) and indirect (25–27) lines of evidence. We find that due to the specific dynamics of underdominant selection maintained by stabilizing selection, BGS can, counterintuitively, lead to an increase in the genetic variance of quantitative traits.

## Results

### Background Selection in Single Site Models.

#### Neutral alleles.

We begin by considering a diploid population of size *N* and an individual site embedded in a chromosome with a total *M* sites that recombine with rate *r* per site, each producing deleterious mutations (with selection coefficient $s_{b}$) at rate *ν*. We assume selection against these mutations is strong relative to drift (i.e., $2Ns_{b}\gg 1$, so they are incapable of fixing), but weak relative to the total recombination rate (i.e., $s_{b}\ll Mr$). For now, we suppose this site is neutral, but will relax this shortly.

With these assumptions, the impact of BGS can be understood through an “effectively nonrecombining linkage block” approximation (see *SI Appendix*, Text B; 28–30). This model approximates the background as a nonrecombining block of characteristic length Msb=2sbr, a scale set by the balance between the purging of deleterious mutations and the erosion of the haplotype by recombination. Because the expected frequency of the deleterious allele at background sites is νsb, the characteristic block carries an average of

[1] $$\begin{array}{c} \lambda =M_{s_{b}}\frac{\nu }{s_{b}}=2\frac{\nu }{r}. \end{array}$$

deleterious alleles (*SI Appendix*, Eq. **B.4**).

The fate of a focal neutral allele depends on two processes operating on different timescales: the purging of preexisting deleterious mutations on its initial background and the accumulation of new deleterious mutations over time. The first process operates on a timescale of Tsb≈1sb generations, the time required for selection to eliminate a haplotype carrying a single deleterious allele. The second process arises because a focal allele that is *t* generations old remains associated with an unrecombined block of expected length Mt=2rt, which is much larger than $M_{s_{b}}$ for $t\ll T_{s_{b}}$. Newly arising deleterious mutations anywhere on this extended block drag the focal allele toward elimination. The balance between mutation and recombination keeps the expected number of these new mutations constant at *λ*, producing a constant expected fitness drag of $s_{\mathrm{drag}}=\lambda s_{b}$.

**Fig. 1.**
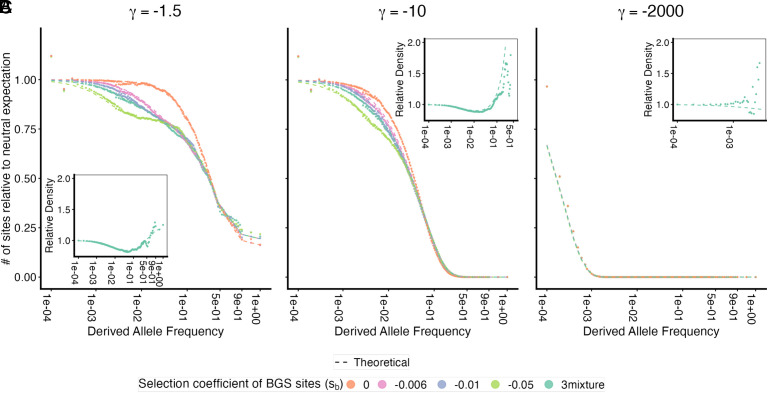
The frequency spectrum of focal selected alleles in the weak mutation limit (λ≪1). The site frequency spectrum observed in simulations (points) are compared to theoretical predictions (lines) for focal alleles under varying strengths of direct selection, assuming a background selection intensity of B≈0.82 (the human average). The site frequency spectrum is scaled relative to the standard neutral expectation (θq). Focal alleles are (*A*) nearly neutral (2Ns=−1.5), (*B*) moderately deleterious (2Ns=−10), and (*C*) strongly deleterious (2Ns=−2,000). Different colors represent simulations where the same total reduction in effective population size (*B*) is achieved using different selection coefficients or a distribution with an equal mixture of all three for the background deleterious mutations ($s_{b}$). The dashed lines represent the approximation developed in Eq. **4** for panels (*A* and *B*), and Eq. **5** for panel (*C*). *Insets* show the relative density of alleles across frequency bins, comparing scenarios with background selection to those without, with theoretical prediction plotted in a dashed line.

If mutation is weak relative to recombination (specifically λ≲12), this fitness drag is negligible compared to selection against individual preexisting deleterious mutations (i.e., $s_{\mathrm{drag}}=\lambda s_{b}\ll s_{b}$). Consequently, the allele’s fate is determined by the preexisting deleterious mutations on its initial background of length $M_{s_{b}}$ before the drag has had time to become relevant. Given this, we can obtain a refined approximation for the classic BGS process that accounts for the frequency-dependent purging of these initial backgrounds. The expected deleterious load on a haplotype block of length $M_{s_{b}}$ at frequency *q* is exponentially suppressed to $\lambda_{q}=\lambda e^{-4Ns_{b}q}$. To reach frequency *q*, a focal allele must therefore reside on a background that carries, on average, $\lambda -\lambda_{q}=\lambda \left(1-e^{-4Ns_{b}q}\right)$ fewer deleterious mutations than a background chosen at random. This purging process reduces the number of alleles that persist to frequency *q* by a factor equal to the survival probability of the background:

[2] $$\begin{array}{c} B\left(q\right)=e^{-\lambda \left(1-e^{-4Ns_{b}q}\right)} \end{array}$$

(*SI Appendix*, Eq. **B.26**), which converges to the “reduced effective population size” approximation, $B=e^{-\lambda }$, for q≫14Nsb (2, 3). Thus, in the weak mutation limit, the skew in the frequency spectrum mirrors the exponential suppression of frequencies among the deleterious alleles responsible for BGS (i.e. $e^{-4Ns_{b}q}$). This approximation can be straightforwardly extended to a distribution over $s_{b}$ by averaging the exponential suppression term:

[3] $$\begin{array}{c} B\left(q\right)\approx e^{-\lambda \left(1-E_{s_{b}}\left[e^{-4Ns_{b}q}\right]\right)} \end{array}$$

(*SI Appendix*, Eq. **B.27**). While these approximations are only formally justified in the limit as λ→0, in practice, we find that they provide a good fit for λ≲12 (see *SI Appendix*, Fig. S1 and Fig. 1 below), which corresponds to B≥0.6, encompassing roughly the full range of background selection effects observed in humans (5, 6).

When mutation is strong relative to recombination (λ≳12), the fitness drag due to newly arising mutations can no longer be neglected and begins to shape the frequency spectrum at frequencies below the classical threshold. Because the drag is constant rather than growing linearly as in the analogous nonrecombining model, it becomes visible to selection on a timescale that is shorter by a factor of 1λ, and consequently distorts the frequency spectrum down to proportionally lower frequencies. In *SI Appendix*, Text B, we extend this perspective to derive a piecewise approximation for the frequency spectrum in the strong mutation regime, analogous to the results of ref. 14 for nonrecombining regions.

#### Directly selected alleles.

Now, suppose the focal site itself produces alleles experiencing additive direct selection with coefficient *s*, and let $\xi \left(q|\gamma \right)=\frac{2}{q(1-q)}\frac{1-e^{-2\gamma (1-q)}}{1-e^{-2\gamma }}$ be the amount of time such an allele spends at frequency *q* in the absence of BGS, where γ=2Ns is the scaled selection coefficient (31, 32).

When direct selection on the focal allele is weak relative to background selection ($\left|\gamma \right|\ll 2Ns_{b}$), it influences dynamics only at high frequencies (q≫14Nsb), where the initial cull of alleles arising on deleterious backgrounds is complete ($B\left(q\right)=e^{-\lambda }$ for q≫14Nsb). This reflects a separation of timescales: Because haplotypes carrying deleterious alleles are rapidly purged, alleles reaching high frequency must have arisen on backgrounds that are free of deleterious mutations over the characteristic length-scale (19, 20). A new, weakly selected mutation is thus expected to spend

[4] $$\begin{array}{cc} \xi_{B}\left(q|\gamma \right) & \approx B\left(q\right)\xi \left(q|\gamma B\right) \end{array}$$

generations at frequency *q* (*SI Appendix*, Eq. **B.30**). Consequently, for deleterious alleles under direct selection weaker than the background intensity, BGS reduces sojourn times at low frequencies but increases them at high frequencies. Notably, this effect persists even for alleles that are strongly selected relative to drift (|γ|≫1), provided they satisfy $\left|\gamma \right|\ll 2Ns_{b}$ (Fig. 1*B*). For beneficial alleles, these effects compound, further suppressing the number of alleles that reach high frequencies (*SI Appendix*, Fig. S2).

When direct selection is strong ($\left|\gamma \right|≳2Ns_{b}$), direct and background selection act on similar timescales. The allele’s fate is determined by its own fitness effect before the background is purged, suggesting the alternative approximation

[5] $$\begin{array}{cc} \xi_{B}\left(q|\gamma \right) & \approx B\left(q\right)\xi \left(q|\gamma \right). \end{array}$$

(*SI Appendix*, Eq. **B.35**). In practice, however, these strongly selected alleles rarely reach the high frequencies where background selection takes effect (q≫14Nsb). Consequently, for the vast majority of variants in this regime, B(q)≈1 and BGS is irrelevant. While we do observe some distortions in the frequency spectrum for q≳14Nsb (Fig. 1*C*), these rare variants contribute minimally to standing variation.

### Phenotypes Under Directional Selection.

#### Heterozygosity and fixation rates at individual sites.

We now consider models of mutation–selection–drift balance (MSDB) for polygenic phenotypes. We analyze these models using a mean-field approach, approximating the evolution of the phenotypic distribution by the average behavior of a single site. The key features of this behavior are captured by two quantities: an allele’s fixation probability and its expected lifetime contribution to heterozygosity.

As shown by ref. 33, these two quantities are intimately related, as the lifetime contribution to heterozygosity is

[6] $$\begin{array}{c} h_{d}\left(\gamma \right)=2\left(\frac{\rho (\gamma )-1}{\gamma }\right), \end{array}$$

where ρ(γ)≈2γ(1−e−2γ) is the expected number of fixations per 2N new mutations. The quantity ρ(γ)−1 gives the “selective fixation rate,” the rate at which fixations are driven or prevented *by* selection. To model the impact of background selection, we employ the standard reduced effective size approximation ($B=e^{-\lambda }$). While Eq. **4** breaks down for B≲0.6, this approximation remains accurate for the heterozygosity provided that $2NBs_{b}\gg 1$—a condition that ensures local effective size reductions are not severe enough to push sites into the interference selection regime (29). This means that our phenotypic results should apply in the presence of much greater effective size reductions than our approximation for the skew.

**Fig. 2.**
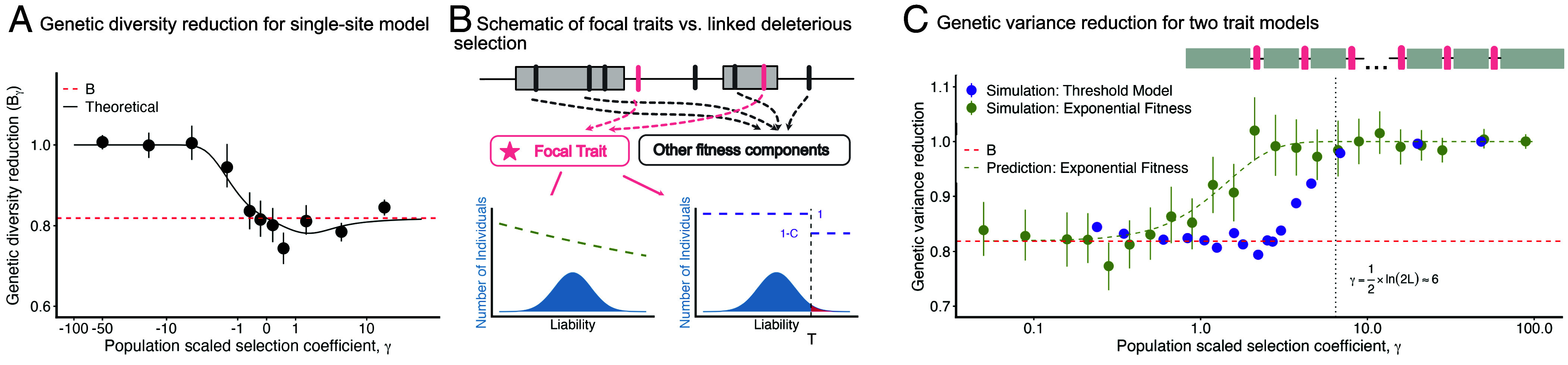
The impact of background selection on genetic variation under directional selection. (*A*) The reduction in genetic diversity for single-site additive directional selection model, represented as the ratio of expected heterozygosity with BGS to that without BGS (hd(γB)hd(γ)), is plotted against the population-scaled selection coefficient (*γ*). Simulation results (points) are shown alongside the theoretical prediction from Eq. **7** (solid line). The horizontal red dashed line indicates the expected diversity reduction for a neutral allele, B≈0.82. (*B*) A schematic depicting the relationship between sites that are causal to a focal trait (pink bars) and linked deleterious mutations that contribute to other fitness components (gray bars), which are responsible for BGS effects. (*C*) We compare the genetic variance reduction ($V_{G,B}/V_{G}$) for the exponential fitness model (green) and the single effect size liability threshold model (purple). Simulation results are plotted as points, and the prediction for the exponential fitness function from Eq. **11** is plotted as a green dashed line. The horizontal red dashed line indicates the expected diversity reduction for a neutral allele, B≈0.82. The vertical dotted line marks γ≈6, which corresponds roughly to pT≈12L given that we use $L=10^{5}$ to simulate. The chromosome structure we used to simulate the threshold model is depicted at *Right Top*, showing trait loci (pink bars) interspersed among BGS sites (gray regions).

Applying this approximation, the reduction in the lifetime contribution to heterozygosity due to BGS is exactly equal to the effect on the selective fixation rate:

[7] $$\begin{array}{c} B_{\gamma }=B\frac{h_{d}\left(\gamma B\right)}{h_{d}\left(\gamma \right)}=\frac{\rho (\gamma B)-1}{\rho (\gamma )-1}, \end{array}$$

where the factor of *B* represents the probability of surviving the initial low-frequency cull, and hd(γB)hd(γ) represents the effect on the lifetime contribution to heterozygosity conditional on surviving this cull (Fig. 2*A*). Notably, this relationship holds even for strongly selected sites (γ≪−1) where the physical mechanism differs. In the regime where direct selection is strong relative to drift but weak relative to background selection ($2Ns_{b}\gg \left|\gamma \right|\gg 1$; Fig. 1*B*), BGS significantly distorts the frequency spectrum: It reduces the number of alleles that survive the initial purge, but allows the survivors to drift to higher frequencies due to weakened effective selection. These opposing effects cancel out, leaving the total heterozygosity unchanged ($B_{\gamma }\approx 1$). Conversely, when direct selection dominates background selection ($\left|\gamma \right|\gg 2Ns_{b}$; Fig. 1*C*), the focal allele is purged by its own fitness cost before the background can be removed. In this limit, BGS is irrelevant, and the heterozygosity is naturally unaffected. We can therefore rely on this reduced local $N_{e}$ approximation to model the underlying allelic dynamics across the full range of selection coefficients.

#### Phenotype model and chromosome structure.

To apply these insights in the context of phenotypic evolution, we assume a simple additive model where each site *ℓ* has two alleles differing by effect $a_{\ell }$. Individual *i*’s trait value is

[8] $$\begin{array}{cc} Z_{i} & =G_{i}+E_{i}=\sum_{\ell }a_{\ell }g_{i\ell }+E_{i}, \end{array}$$

where $g_{i\ell }\in \left\{0,1,2\right\}$ is the genotype count and $E_{i}∼N\left(0,V_{E}\right)$. We assume that causal sites are sufficiently spaced such that their local evolutionary dynamics can be treated independently. Each site is embedded in a recombining background that generates unconditionally deleterious alleles according to the model described above; however, we allow the density of these mutations per unit recombination (i.e. λ=2νr)—and thus the magnitude of the local effective population size reduction (i.e. $B=e^{-\lambda }$)—to vary across causal sites.

#### Exponential selection.

The simplest model of directional selection is one in which fitness declines exponentially with increasing phenotype, i.e., $W\left(Z_{i}\right)\propto e^{-Z_{i}/\eta }$ (Fig. 2*B*). In this model, sites evolve independently with an approximately constant directional selection coefficient $s\approx a\eta^{-1}$, corresponding to a scaled selection coefficient $\gamma =2Ns\approx 2Na\eta^{-1}$ (*SI Appendix*, Text C).

At mutation–selection–drift equilibrium, fixations of trait-increasing and trait-decreasing mutations reach a detailed balance (34–36). Among sites with scaled selection coefficient *γ*, the fraction of sites fixed for the trait-increasing (deleterious) allele is determined by the ratio of fixation rates,

[9] $$\begin{array}{c} p^{+}\left(\gamma \right)=\frac{\rho (-\gamma )}{\rho (\gamma )+\rho (-\gamma )}=\frac{1}{1+e^{2\gamma }}. \end{array}$$

The resulting genetic variance reflects a combination of contributions from alleles under positive and negative selection

[10] $$\begin{array}{c} V_{G}=\theta \left[p^{-}\left(\gamma \right)h_{d}\left(-\gamma \right)+p^{+}\left(\gamma \right)h_{d}\left(\gamma \right)\right]=a^{2}\frac{\theta }{\gamma }b\left(\gamma \right), \end{array}$$

where b(γ)=ρ(γ)−ρ(−γ)ρ(γ)+ρ(−γ)= tanh(γ) measures the asymmetry of the mutational input at equilibrium.

Because of the independence among sites, the impact of background selection on each site is captured entirely by the local reduction in effective population size. This leads to a rescaled fixation asymmetry $p^{+}\left(\gamma B\right)={\left(1+e^{2\gamma B}\right)}^{-1}$, driving a large shift in the mean phenotype. For weakly selected sites, this shift is much larger than the scale of the standing genetic variation (i.e., $\left({\overset{¯}{G}}_{B}-\overset{¯}{G}\right)/\sqrt{V_{G}}\gg 1$), resulting in a significant decline in the mean fitness of the population (*SI Appendix*, Text C).

The impact on the variance follows from the relationship between heterozygosity and fixation rates established in Eq. **7**. Specifically, the variance is reduced by a factor

[11] $$\begin{array}{cc} \frac{V_{G,B}}{V_{G}} & =\frac{b(\gamma B)}{b(\gamma )}=\frac{\tanh(\gamma B)}{\tanh(\gamma )} \end{array}$$

(Fig. 2*C*; 37). This reduction is greatest for effectively neutral alleles ($V_{G,B}/V_{G}\approx B$ for γ≪1) but weakens as selection strength increases, vanishing entirely for strongly selected sites (γ≫1). This fading reflects the fact that strong negative selection purges alleles faster than BGS can remove the background, rendering the reduction in $N_{e}$ irrelevant. Because the selection coefficient *s* in this model is fixed by the fitness function parameter *η*, it is unaffected by the shift in the mean phenotype. As a result, all sites respond to their local BGS effects independently, and the effect on the phenotype in any given case is simply found by averaging these effects over the joint distribution of effect sizes and local effective size reductions.

#### Threshold epistasis.

In this model, fitness depends on whether the phenotype exceeds a threshold, *T*. Individual *i*’s relative fitness is $W\left(Z_{i}\right)\propto 1-1\left[Z_{i}>T\right]C$, where *C* is the cost of exceeding the threshold and $1[Z_{i}>T]$ indicates whether $Z_{i}>T$ (Fig. 2*B*). We treat this as a model of complex disease, where $Z_{i}$ represents an individual’s liability (21). Equivalent threshold models have appeared in other contexts, where the phenotype is simply termed “fitness potential” (38, 39). We adopt the complex disease framework here, though our results could easily be applied in other contexts by relabeling terms.

#### A single effect size and a single B value.

The threshold model differs from the exponential model in two fundamental ways, which are easiest to see if we first assume a single effect size, *a*, and constant local reduction in the effective population size, *B*, across sites. First, the threshold imposes strong epistasis: A site’s selection coefficient depends on the variation at all other sites. However, because the background phenotypic variation smoothes out the sharp epistasis of the threshold, the marginal effect of an allele on fitness is nonetheless constant, allowing individual sites to evolve as if under additive selection. If the liability effect *a* is small relative to the variance in liability ($a\ll \sqrt{V_{P}}$), this dependency is mediated solely by the liability density at the threshold, f(T):

[12] $$\begin{array}{c} s=\delta C\approx af(T)C, \end{array}$$

where δ≈af(T) is the site’s effect on disease risk (21).

Second, because the threshold has a fixed position along the liability continuum, it determines the fraction of sites fixed for the liability-increasing allele p+(γ)≈pT=T2La. Consequently, the threshold position determines the scaled selection coefficient via the relationship between the fixation asymmetry, $p^{+}\left(\gamma \right)$, the fixation rates, $\rho \left(\gamma \right)$, and *γ* (Eq. **9**). In a single *a* model, we can solve directly:

[13] $$\begin{array}{c} \gamma \approx \frac{1}{2}\ln\frac{1-p_{T}}{p_{T}} \end{array}$$

Ref. 21. Combining this with the relationship between threshold density f(T) and the selection coefficient (Eq. **12**), equilibrium is established when the mean liability is positioned at a distance just below the threshold, such that $f\left(T\right)\approx \frac{1}{4NCa}\ln\frac{1-p_{T}}{p_{T}}$. Because sites evolve under additive selection, genetic variance depends on *γ* exactly as in the exponential model (Eq. **10**); however, *γ* is now determined by the threshold position rather than the rate of fitness decline.

In the threshold model, strong selection against individuals with liability greater than *T* prevents the large shift in the mean that occurs in the exponential model. Consequently, the system must maintain the equilibrium fraction of liability increasing fixations ($p^{+}\left(\gamma \right)\approx p_{T}$). As a result, BGS must reduce the selective fixation rates for liability increasing and liability decreasing mutations by the same fraction. This constraint requires that the reduction in genetic variance must arise *solely* from the initial culling of new mutations (i.e., the factor *B*). Recall from Eq. **7** that the total impact of BGS is the product of this initial cull and the change in the per-mutation contribution to variance ($h_{d}\left(\gamma_{B}\right)/h_{d}\left(\gamma \right)$). Therefore, for the system to maintain equilibrium via the initial cull alone, the per-mutation contribution must remain unchanged. This implies that the scaled selection coefficient itself must be invariant, i.e. $\gamma_{B}=\gamma$, which then satisfies the initial requirement that the selective fixation rates are modified by the same factor, i.e.

[14] $$\begin{array}{c} \frac{B\left(\rho (\gamma_{B})-1\right)}{\left(\rho (\gamma )-1\right)}=\frac{B\left(\rho (-\gamma_{B})-1\right)}{\left(\rho (-\gamma )-1\right)}. \end{array}$$

The system satisfies this requirement through a global compensation mechanism. In response to the local increase in genetic drift, the mean liability shifts slightly closer to the threshold, increasing the threshold density to $f_{B}\left(T\right)\approx f\left(T\right)/B$. This global adjustment increases the unscaled selection coefficients ($s_{B}\approx s/B$), which offsets the local reduction in the effective population size (N→NB):

[15] $$\begin{array}{c} \gamma_{B}=2N_{B}s_{B}=2\left(NB\right)\frac{s}{B}=2Ns=\gamma . \end{array}$$

Because *γ* is invariant, the per-mutation variance $h_{d}\left(\gamma \right)$ remains unaffected, as predicted. The total reduction in genetic variance is therefore driven entirely by the reduced supply of mutations (θ→θB), resulting in a simple, *γ*-independent reduction in genetic variance:

[16] $$\begin{array}{c} \frac{V_{G,B}}{V_{G}}=B. \end{array}$$

**Fig. 3.**
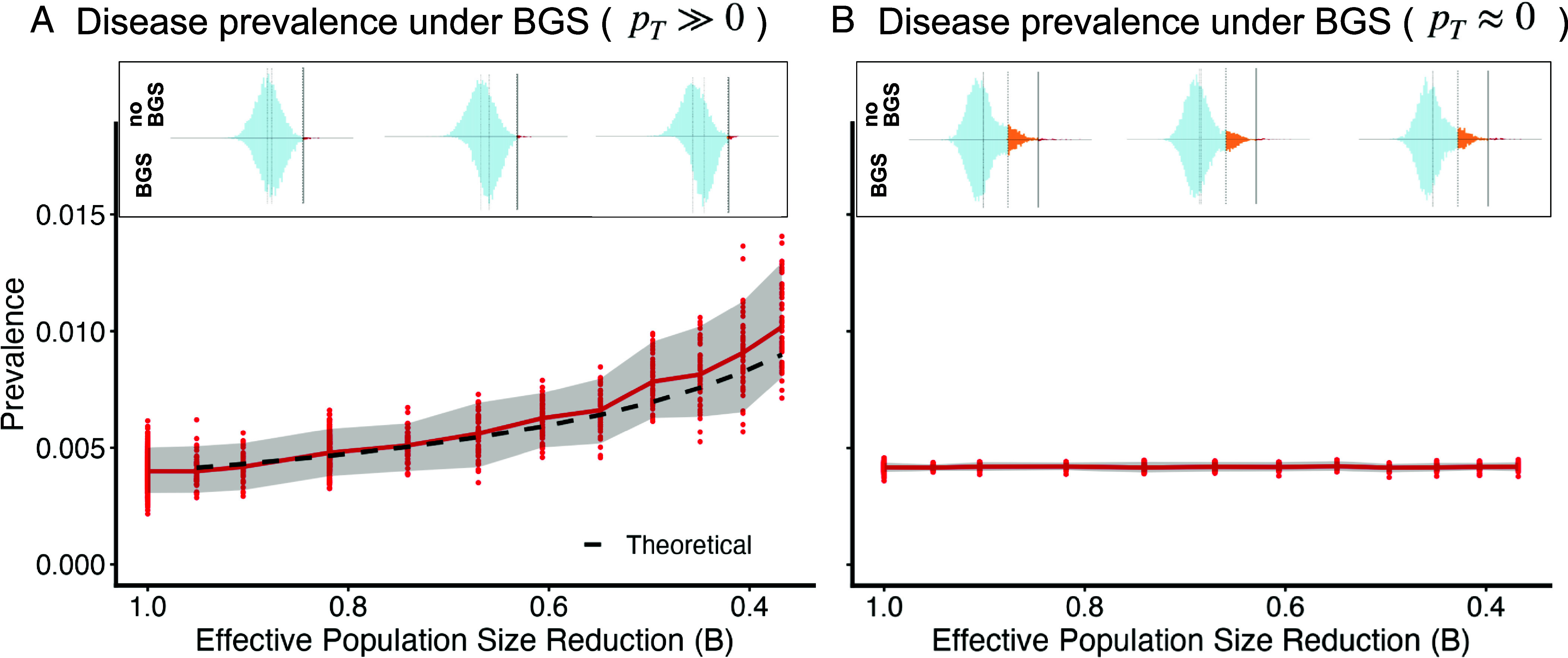
Disease prevalence under BGS is shown for scenarios where (*A*) the equilibrium fraction of liability increasing fixations is large ($p_{T}\gg 0$), vs. (*B*) scenarios where liability increasing fixations are absent ($p_{T}\approx 0$). The prediction from Eq. **17** is plotted in dashed line. In the *Inset*, the liability distribution in one simulation is plotted comparing BGS to no BGS at background selection intensities of approximately B≈(0.95,0.67,0.45) from left to right.

Thus, the threshold model avoids the large decrease in mean fitness characteristic of the exponential model through a global compensation of selection coefficients that maintains the mean phenotype. This compensation impacts the liability distribution by simultaneously reducing the variance ($V_{G}\to BV_{G}$) and increasing the threshold density (f(T)→f(T)/B) (Fig. 2*C* and *SI Appendix*, Figs. S3 and S7). These two effects combine to increase the disease prevalence (Fig. 3*A*) by a factor of approximately

[17] $$\begin{array}{c} \frac{{\overset{¯}{R}}_{B}}{\overset{¯}{R}}\approx \frac{\sqrt{1-h^{2}\left(1-B\right)}}{B}, \end{array}$$

where $h^{2}$ is the heritability of liability without BGS (We also used simulations to explore whether the skew in the frequency spectrum impacts the prevalence, but observed no effect; see *SI Appendix*, Text D and Fig. S4).

However, both the invariance of *γ* and the increase in prevalence depend entirely on the global compensation mechanism, which is itself predicated on the existence of deleterious fixations to balance (i.e., $p_{T}>0$; Eq. **13**). This assumption breaks when the threshold is so low that $p_{T}$ is driven to effectively zero. In a genome with *L* contributing sites, this occurs roughly when the expected number of fixed deleterious alleles drops below one (pT≲12L), corresponding to a critical selection coefficient of $\gamma \approx \frac{1}{2}\ln\left(2L\right)$. If *γ* exceeds this value, there are no deleterious fixations left to maintain. The global compensation mechanism shuts down, *γ* is no longer forced to be invariant, and the system reverts to standard local dynamics. Consequently, in this strong selection regime, the distinctive signatures of the threshold model disappear: The *γ*-independent variance reduction fades away (Fig. 2*C*), and the increase in disease prevalence predicted by Eq. **17** does not occur (Fig. 3*B*).

#### Variation in effects and variation in B values.

The threshold model described above assumes a single effect size and constant $N_{e}$ reduction, allowing for a straightforward population size rescaling. However, if effect sizes (*a*) and local $N_{e}$ reductions (*B*) vary across sites, the dynamics are complicated by the fact that all sites remain coupled by a single threshold density, f(T). The BGS-driven increase in f(T) must therefore represent a global compromise set by the varying impacts of local effective size reductions across sites. This single compensatory change then impacts the selection coefficients of nearly all sites. To capture these dynamics, we must first understand the global compensatory evolution of the threshold density, f(T), and then resolve how this global change impacts sites with different effect sizes and local effective size reductions.

For simplicity, suppose *a* and *B* vary independently across sites with densities $g_{a}\left(a\right)$ and $g_{B}\left(B\right)$. Then, the equilibrium threshold density, $f\left(T\right)$, must solve:

[18] $$\begin{array}{c} p_{T}=\int g_{B}\left(B\right)\int g_{a}\left(a\right)\frac{a}{\overset{¯}{a}}p^{+}\left(2NC\cdot f\left(T\right)\cdot aB\right)\;\text{d}a\text{d}B. \end{array}$$

**Fig. 4.**
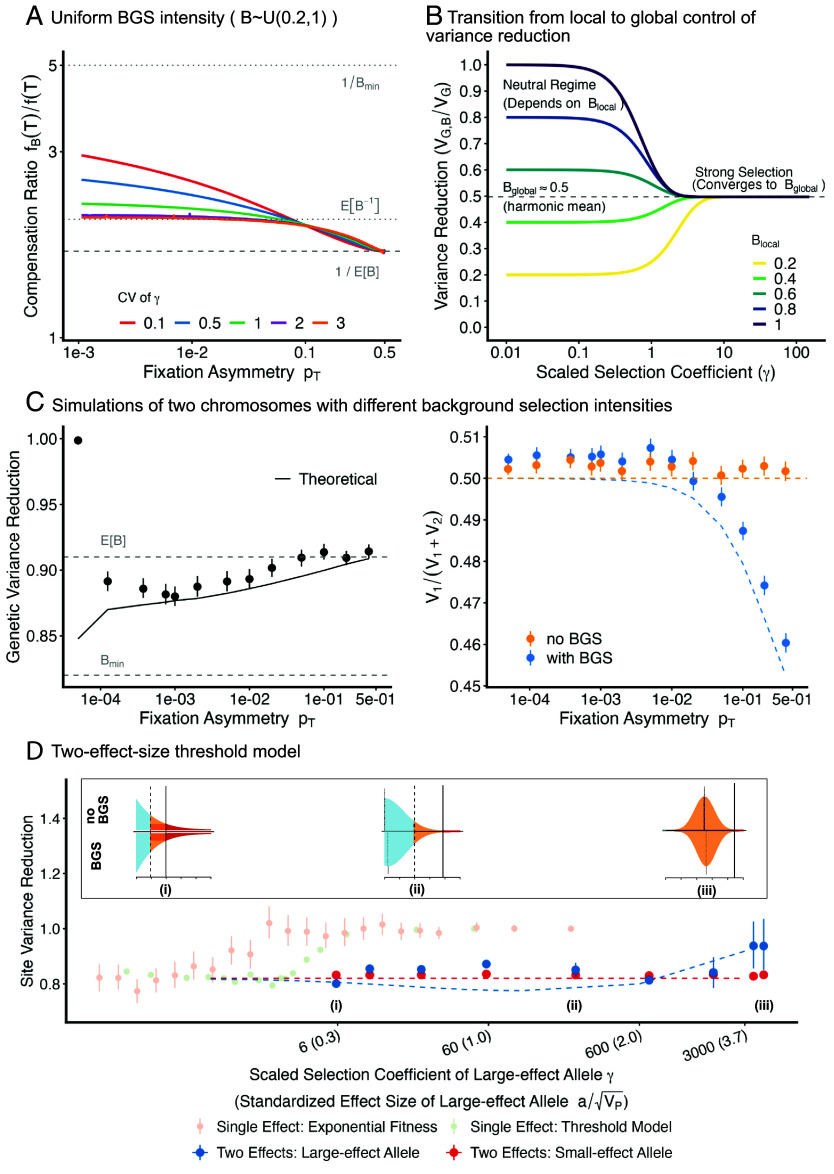
Global coupling of background selection effects in the liability threshold model. (*A*) The threshold density ratio, $f_{B}\left(T\right)/f\left(T\right)$, plotted against the fixation asymmetry ($p_{T}$) for different coefficients of variation (CV) of the effect size distribution. Curves give numerical solutions to Eq. **18**, assuming a Gamma distribution for effect sizes and a uniform distribution of local B values, B∼U(0.2,1). Horizontal dashed lines indicate theoretical limits derived in the text. (*B*) The variance reduction due to BGS as a function of scaled selection coefficient *γ* and local *B* value (Eq. **22**), assuming B∼U(0.2,1) and the high CV limit so that Bglobal=1E[B−1]. Curves illustrate the transition from dependence on $B_{\mathrm{local}}$ to the global average governed by $B_{\mathrm{global}}$. (*C*) We simulated a single-effect-size model with causal sites distributed across two chromosomes. We compare simulations in which chromosome one experiences an effective size reduction of B≈0.82 while the other experiences no BGS. The *Left* panel shows the reduction in total genetic variance across both chromosomes in the simulations with BGS relative to those without. The solid black line plots the numerical solution of Eq. **18**. The *Right* panel reports the fraction of genetic variance contributed by chromosome one in the presence (blue) and absence of BGS (orange). The theoretical prediction is obtained from Eq. **22**. (*D*) Simulations with one set of sites having small effect sizes and being weakly selected, and the other set of sites having large effects and being strongly selected, with all sites sharing the same local *B*. We varied the effect sizes of the large-effect alleles and measure the effect of background selection on the contribution to variance. The x axis measures the population scaled selection coefficient *γ* of the large effect allele, while the effect of the large effect allele relative to the scale of liability variance is given in parentheses. The theoretical predictions are obtained by solving the two effect model numerically, see *SI Appendix*, Text E. To compare the two-effect size results with the single-effect model, genetic variance reductions for the exponential model and the single-effect-size threshold model are shown in light pink and light green. (*i*)–(*iii*) illustrate how the risk effect size, *δ*, for the large-effect-size allele changes with BGS depending on its effect size, driving this effect (recall that s≈δC). The orange shaded region illustrates the proportion of the population pushed across the threshold by the allele, i.e. *δ*. (*i*) The effect size is still relatively small, and the risk effect size can be linearly approximated via the dark orange rectangle (i.e., Eq. **12**). BGS increases the area of this rectangle by a factor of 1B. (*ii*) This linearity breaks down, and BGS results in an increase of *δ* by a factor of more than 1B. (*iii*) When the effect size is large enough to push the entire population across the threshold, BGS no longer has any impact on *δ*, and therefore no effect on selection coefficients.

In *SI Appendix*, Text F, we find analytical approximations for this integral in tractable limits assuming a gamma distribution on effect sizes $g_{a}\left(a\right)$. Here, we summarize the resulting behaviors by categorizing the distinct regimes controlled by the value of $p_{T}$ (Fig. 4*A*). We first consider the effectively neutral limit ($p_{T}\approx \frac{1}{2}$) before examining the transition toward the strong selection limit ($p_{T}\to 0$). Notably, as selection strengthens, the solution depends heavily on the shape of the effect size distribution; we therefore contrast distributions with a low coefficient of variation (CV) against those with a high CV (e.g., “L-shaped” distributions) to illustrate how the tail of the distribution shapes the global compensation.

When $p_{T}\approx \frac{1}{2}$, neutral dynamics and local $N_{e}$ effects dominate. The global compensation required to maintain equilibrium is set by the arithmetic mean of *B*:

[19] $$\begin{array}{c} \frac{f_{B}\left(T\right)}{f\left(T\right)}\approx \frac{1}{E\left[B\right]}. \end{array}$$

However, because most sites are effectively neutral, they are insensitive to this global change in f(T). Their dynamics are instead dominated by their local $N_{e}$ reduction, meaning their individual variance reductions simply depend on their local *B* value.

In the limit where we decrease $p_{T}$ toward zero, the solution to Eq. **18** depends heavily on the shape of the effect size distribution, $g_{a}\left(a\right)$. If $g_{a}\left(a\right)$ has a low coefficient of variation (CV), so that the effect sizes are tightly clustered around the mean, then the bulk of sites transition into the strong selection regime simultaneously as $p_{T}\to 0$. In this limit, the fixation asymmetry decays exponentially (i.e., $p^{+}\left(\gamma \right)\approx e^{-2\gamma }$). The fixed liability burden at equilibrium is therefore sustained almost entirely by the few sites where genetic drift is strong enough to overpower selection—specifically, those with the smallest scaled selection coefficients. In a model where effect sizes are all roughly equal to one another, these sites are the ones with the strongest local BGS effects (i.e. the smallest *B* values). Consequently, the global compensation converges to depend on the minimum *B* value in the genome:

[20] $$\begin{array}{c} \frac{f_{B}\left(T\right)}{f\left(T\right)}=\frac{1}{B_{\min}}. \end{array}$$

Thus, in this limit, the specific local $N_{e}$ reductions at most sites become irrelevant, as the global equilibrium is determined by the region experiencing the strongest BGS.

However, as in the single-effect-size case, this compensation mechanism shuts down when $p_{T}≲\frac{a_{\min}}{2\overset{¯}{a}L}\approx \frac{1}{2L}$. At this point, even sites with the minimum effect size, $a_{\min}$, can no longer be fixed for the deleterious allele. Thus, for a fixed low-CV distribution, decreasing $p_{T}$ from $\frac{1}{2}$ to 0 causes the ratio fBTfT to first increase from $E{\left[B\right]}^{-1}$ toward $\frac{1}{B_{\min}}$, and then abruptly drop to 1 as $p_{T}$ approaches $\frac{1}{2L}$ (Fig. 4*C*).

Alternatively, if the effect size distribution has a high coefficient of variation (CV)—corresponding to the “L-shaped” limit of the gamma distribution—sites will be spread across all selective regimes simultaneously, but with the bulk of sites remaining in the effectively neutral regime near zero. The resulting global compensation factor, fBTfT, must represent a compromise between the limits set by the different regimes: The large mass of neutral sites pulls the compensation toward the neutral limit ($E{\left[B\right]}^{-1}$), while the exponential sensitivity of the sites in the tail pulls it toward the strong selection limit ($B_{\min}^{-1}$). In *SI Appendix*, Text G, we show that in this high-CV limit, this compromise converges to the reciprocal of the harmonic mean *B* value:

[21] $$\begin{array}{c} \frac{f_{B}\left(T\right)}{f\left(T\right)}=E\left[B^{-1}\right], \end{array}$$

which satisfies 1EB≤EB−1≤1Bmin for any distribution $g_{B}$. In Fig. 4*A*, we illustrate the convergence to this limit by numerically solving Eq. **18** assuming that the local effective size reductions are uniformly distributed between 0.2. and 1.

In contrast to the low-variance case, this global compensation persists even as $p_{T}\to 0$. This difference stems from the high density of small-effect variants in distributions with a mode at zero (e.g., the high-CV Gamma). In the low-CV case, all sites have approximately the same effect ($a\approx \overset{¯}{a}$), so they all transition into the strong selection regime simultaneously, causing the compensation mechanism to shut down abruptly. In the high-variance case, there is no such simultaneous shutdown. As the threshold density f(T) increases, sites with large effects are indeed pushed into the strong selection regime, where they stop contributing to the fixed liability burden. However, the mode at zero ensures a continuous reservoir of sites with even smaller effects. As sites with larger effects are all pushed into the strong selection regime, the burden of maintaining the total equilibrium fixation asymmetry dictated by the threshold shifts to these smaller effect sites, which—precisely because of their small effects—remain in the weakly selected regime where they are sensitive to changes in local effect size (*SI Appendix*, Text G).

While the specific result in Eq. **21** arises from the power-law tail of the high-CV gamma distribution, the broader insight is that in biologically realistic scenarios—where both effect sizes (*a*) and local *B* values vary—the phenotype is shaped by a combination of two forces: a single global compensation factor and site-specific local BGS effects. The total impact on any given site depends on its selective regime. For a site with scaled selection coefficient *γ* and local $N_{e}$ reduction $B_{\mathrm{local}}$, the total reduction in genetic variance is given by

[22] $$\begin{array}{c} \frac{V_{G,B}}{V_{G}}=B_{\mathrm{global}}\frac{\tanh\left(\gamma \frac{B_{\mathrm{local}}}{B_{\mathrm{global}}}\right)}{\tanh\left(\gamma \right)}, \end{array}$$

where Bglobal=f(T)fB(T) is the global compensation factor determined by the genome-wide distributions of effects and *B* values (e.g., in the high-CV gamma limit, $B_{\mathrm{global}}$ is the harmonic mean, 1EB−1). Thus, effectively neutral sites are governed by their local effective size reductions (VG,BVG≈Blocal), while strongly selected sites are pinned to the global average (VG,BVG≈Bglobal), regardless of the local *B* value. We illustrate this transition in Fig. 4*B* for the same uniform distribution of local effective size reductions used in Fig. 4*A*.

Notably, this demonstrates that BGS reduces genetic variance even at sites under very strong selection. Unlike single-effect models where global compensation shuts down at the pT→12L boundary, in high-variance models compensation persists, subjecting strongly selected sites to a variance reduction of order $B_{\mathrm{global}}$. This effect has limits, however, as the approximation s≈af(T)C breaks down for very large effects (21). For effects comparable to the liability SD ($a∼\sqrt{V_{P}}$), nonlinearity can drive reductions greater than $B_{\mathrm{global}}$. As *a* increases further, however, carriers cross the threshold regardless of their background liability, and the strength of selection becomes determined solely by the fitness cost (s≈C), independent of f(T), and the global effect on the selection coefficients fades. Notably, this limit is determined by the fitness cost of exceeding the threshold, rather than population-genetic constraints on fixation dynamics. We illustrate these effects in Fig. 4*D* by comparing numerical solutions (*SI Appendix*, Text E) with simulations.

Thus, in realistic high-variance models, BGS reduces genetic variance even at strongly selected sites. The wide distribution of effects ensures that global compensation ($B_{\mathrm{global}}$) remains active, coupling these sites to the genome-wide background. This contrasts with single-effect models, where sites become immune to BGS once compensation shuts down at the pT→12L boundary.

### Stabilizing Selection.

As we have shown, fitness epistasis in the form of threshold selection alters the impact of BGS via a global compensation mechanism, which is necessary to maintain the specific fixation asymmetry imposed by the threshold. We now turn to a model of stabilizing selection, defined by a Gaussian fitness function with an optimum phenotype of $L\overset{¯}{a}$ (i.e., $W\left(Z_{i}\right)=e^{\frac{-{\left(Z_{i}-L\overset{¯}{a}\right)}^{2}}{2\omega^{2}}}$; 40). This model contrasts with the threshold case in two key ways. First, it ensures mutational symmetry at equilibrium, eliminating the net fixation asymmetry that drove the global coupling in the threshold model. Second, the local dynamics differ: This form of epistasis causes individual alleles to evolve as if underdominant, with a selection coefficient $t\approx \frac{a^{2}}{2\omega^{2}}$ against heterozygotes (41, 42). This allows us to isolate the impact of BGS on these purely local dynamics.

Defining the scaled coefficient of underdominance as τ=2Nt, ref. 43 showed that the expected lifetime contribution to heterozygosity is

[23] $$\begin{array}{c} h_{u}\left(\tau \right)\approx 2\int_{0}^{\frac{1}{2}}x\left(1-x\right)\Psi \left(x|\tau \right)dx=2\frac{D_{+}\left(\sqrt{\frac{\tau }{2}}\right)}{\sqrt{\frac{\tau }{2}}},is \end{array}$$

where $D_{+}\left(y\right)$ is the Dawson function, and Ψ(x|τ)=2e−2τx(1−x)x(1−x) is the sojourn time at minor allele frequency *x*. The behavior of this function reveals a crucial deviation from simple directional selection. While $h_{u}\left(\tau \right)$ converges to the directional result $h_{d}\left(-\tau \right)$ at the extremes (τ≪1 and τ≫1), it is significantly elevated in the intermediate regime (1≲τ≲30). In this intermediate regime, alleles that drift to intermediate frequency “slow down” and persist longer than predicted by negative selection alone, causing the ratio $h_{u}\left(\tau \right)/h_{d}\left(-\tau \right)$ to peak at ≈1.29 when $\tau^{*}\approx 4.42$ (*SI Appendix*, Figs. S8 and S9).

Applying the reduced effective size approximation ($N_{e}\to N_{e}B$), the modification to heterozygosity is given by

[24] $$\begin{array}{c} B_{\tau }=B\frac{h_{u}\left(\tau B\right)}{h_{u}\left(\tau \right)}=\sqrt{B}\frac{D_{+}\left(\sqrt{\frac{\tau B}{2}}\right)}{D_{+}\left(\sqrt{\frac{\tau }{2}}\right)}. \end{array}$$

This equation demonstrates that BGS does not simply reduce variance by a factor of *B* or cancel out as it does under strong directional selection. Rather, for some combinations of *τ* and *B*, the increased rate of drift leads sites that would otherwise be held at low frequencies to drift up to higher frequencies where the selection against them is weaker, allowing them to continue drifting even further. The increased contribution to heterozygosity conditional on surviving the low frequency cull due to BGS therefore overcompensates for the heterozygosity it eliminates.

**Fig. 5.**
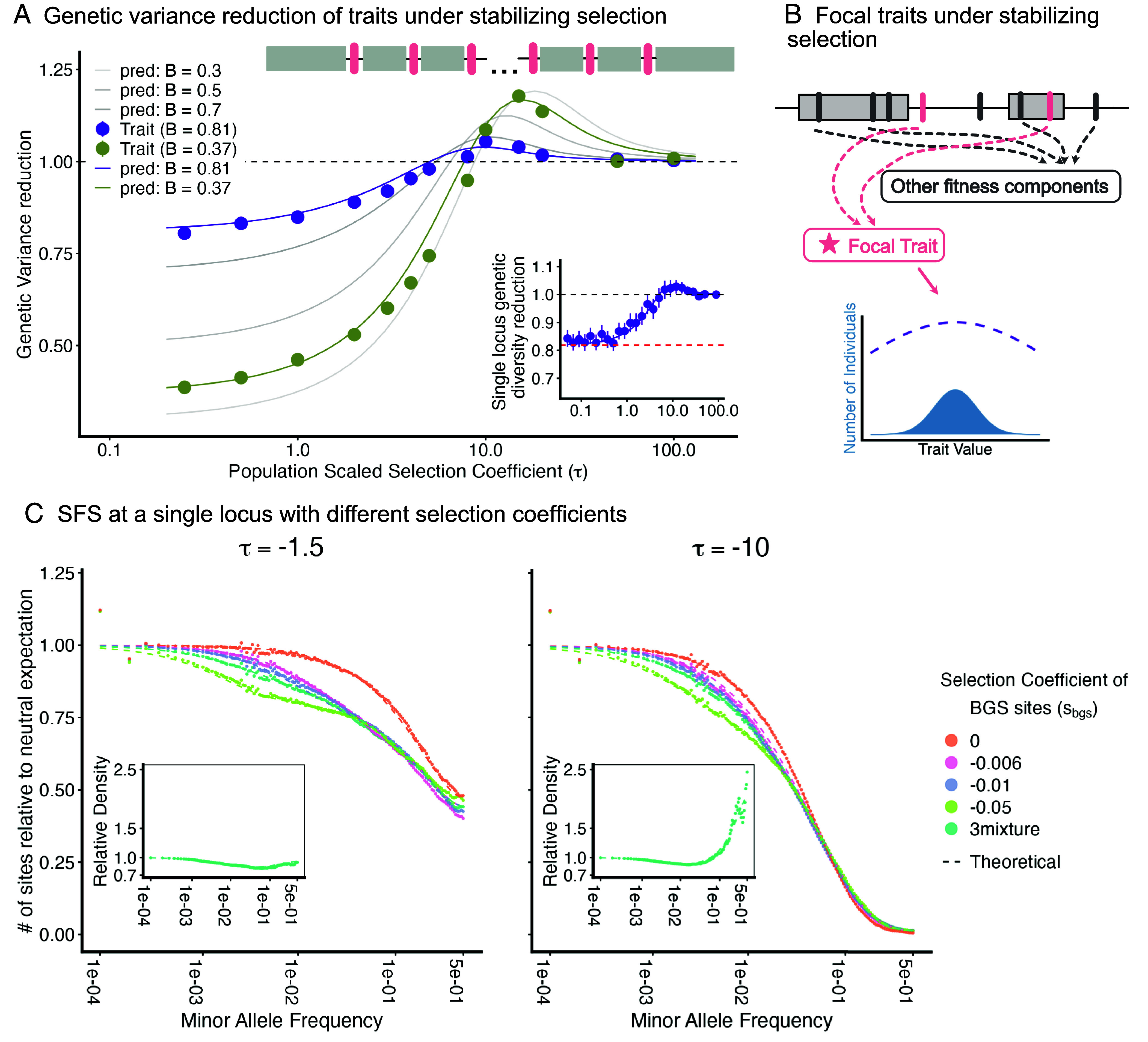
Stabilizing selection. (*A*) We simulate traits with a single effect size under stabilizing selection with two different effective population size reductions. For each effective size reduction, we plot the change in genetic variance against the population-scaled selection coefficient ($\tau =2N\frac{a^{2}}{2\omega^{2}}$). The chromosome structure used for simulation is depicted in the *Top Right*, showing trait loci interspersed among BGS regions. Simulation results (points) are compared with the theoretical prediction, obtained from Eq. **23**, with the B values estimated from the diversity reduction of neutral sites simulated in the same genome. The *Inset* shows the genetic diversity of underdominant alleles in single locus simulations with B=0.82, the human average. (*B*) Schematic describing the relationship between sites contributing to focal traits (pink bars) and linked deleterious mutations (gray bars). (*C*) The site frequency spectrum observed in simulation (points) are compared to the theoretical predictions (dashed lines) for focal selected sites under weak underdominant selection (τ=−1.5, *Left*) and stronger underdominant selection (τ=−10, *Right*). Different colors represent different selection coefficients or a distribution with an equal mixture of all three at the background mutations ($s_{b}$). *Insets* show the relative density of alleles at each frequency bin with background selection vs. no background selection.

This variance inflation is maximized when BGS shifts alleles from the strong selection regime down to the intermediate regime where the impact of underdominance is most pronounced ($\tau^{*}\approx 4.42$). For strong BGS (B≪1), this peak net increase occurs at τ≈τ∗B and converges to the theoretical maximum of ≈1.29, while the range of affected sites scales with 1/B. Fig. 5*A* shows that multilocus simulations of stabilizing selection faithfully replicate these single-site predictions.

In Fig. 5 *C* and *D*, we apply the frequency-dependent factor $B\left(q\right)$ from Eq. Eq. **2** and the rescaling τ→τB to the frequency spectrum for underdominant sites for τ=−1.5 and 10 under modest background selection (i.e., B=0.83, the human average). In this regime, the rescaling of *τ* is not dramatic enough to move sites from the strong into the weak selection regime, so the effects on the frequency spectrum are modest. In contrast, under larger effective population size reductions, as BGS moves sites fully between regimes, the inflation of the frequency spectrum at intermediate frequencies becomes substantial, although here our simple approximation for the skew in the low frequency regime breaks down (*SI Appendix*, Fig. S10).

## Discussion

In this paper, we have used a combination of analytical theory and simulations to study the impacts of background selection in recombining genomes, both at the level of individual variants and the level of phenotypes, in several long-studied models.

### Background Selection and the Frequency Spectrum in Recombining Genomes.

While the impact of background selection on aggregate diversity is often well-approximated by a simple reduction in effective population size, characterizing the full shape of the site frequency spectrum in recombining genomes presents a more difficult challenge. Here, we describe an effectively nonrecombining block approximation (28–30) for the classic model of background selection with recombination by defining the characteristic length scale of the process, and demonstrate that this approximation recovers classic results previously derived by summing contributions across many loci in pairwise models (2, 3). This reframing allows us to make progress in understanding the impact of BGS on the skew in the frequency spectrum in recombining genomes and to derive a simple expression for the effect that is accurate in the weak mutation regime which characterizes humans (i.e. where λ=2νr≲12).

As described in the Results, the shrinking block perspective also reveals a key difference in dynamics between recombining and nonrecombining genomes: In the recombining case, newly arising deleterious mutations far beyond the characteristic length $M_{s_{b}}$ exert a constant fitness drag, rather than the linearly growing deficit that characterizes the analogous nonrecombining model (14). In *SI Appendix*, Text B, we develop this perspective further, mapping the dynamics onto the nonrecombining model and extending the piecewise expression for the frequency spectrum skew in the strong mutation regime (λ≫1) to recombining genomes (*SI Appendix*, Fig. S6). As a consequence of the constant drag, the frequency at which substantial skew begins to accumulate is lowered by a factor of 1λ relative to a nonrecombining model with the same local reduction in effective size (*SI Appendix*, Eq. **B.18**), and the blocks eliminated by this process are longer than the characteristic length $M_{s_{b}}$ by a factor of *λ* (*SI Appendix*, Eq. **B.12**).

It remains a priority for future work to develop an accurate analytical or numerical description of the frequency spectrum for the λ≳12 regime where the accumulation of new deleterious mutations is important. For 12≲λ≲1, progress might be made by assuming the focal allele accumulates at most one additional deleterious mutation within the characteristic length $M_{s_{b}}$, though this approximation would clearly fail for larger *λ*. For the λ≫1 case, the shrinking block perspective is promising: The impact on an allele at frequency *q* could be described by a characteristic length $M_{q}={\left(Nrq\right)}^{-1}$, treating the region as a nonrecombining chromosome of that length. While this approach requires a more robust solution for the frequency spectrum in nonrecombining genomes, bridging the intermediate regime where both preexisting and newly arising mutations play a role in shaping the dynamics, we hope these insights can help provide a pathway toward that solution.

### Models of Phenotypic Selection and Linkage in Recombining Genomes.

In our phenotypic analyses, we assume $2NBs_{b}\gg 1$, ensuring that the skew in the frequency spectrum is fully “priced in” at relatively low frequencies. This permits us to assume that BGS preserves the standard functional forms of heterozygosity and fixation rates under Wright–Fisher diffusion, allowing us to apply classic effective population size rescaling in our phenotypic models while retaining the standard diffusion forms. However, the relationship between an allele’s contribution to heterozygosity and its fixation rate expressed in Eq. **6** is more general than the diffusion limit, as it simply represents an averaging of Fisher’s fundamental theorem over an allele’s transit through the population (41, 44). Consequently, the behaviors we describe for directional models are expected to be extremely general, and should equally apply in models that relax the strong selection assumption (29, 44, 45). Under the threshold model in particular, introducing essentially any form of linked selection should force the population distribution to evolve such that the selective fixation rates in both directions are reduced by the same factor. Even if the form of linked selection alters the functional form of the fixation rate, a reduction in the selective fixation rate by a given multiplicative factor must imply a corresponding reduction in heterozygosity by the same factor, independent of the underlying details (although changes in the form of the fixation rate could alter the precise form of *B* value averaging that drives the global effect).

That said, different models of linked selection nonetheless result in different relationships between fundamental parameters and patterns of genetic diversity, and there remains considerable uncertainty about the underlying source of linked selection signals (5, 6). While we have primarily focused on understanding the impact of BGS on the equilibrium evolution of complex traits, it is also interesting to ask the converse question: What forms of phenotypic variation underlie linked selection signals that have been measured empirically, and what model of selection applies to the underlying loci? This is particularly interesting to consider in light of arguments for the ubiquity of stabilizing selection (22, 26, 27).

Intuitively, alleles with effects large enough to fall into the strong selection regime (i.e., $\tau \approx 2N\frac{a^{2}}{2\omega^{2}}≳30$) should impact linked sites precisely as predicted by classic BGS theory, because their local dynamics mirror the standard strong selection model. A more complex question is what effect we should expect from loci falling into the intermediate weak selection regime, where underdominant dynamics become important. These loci are more likely to drift to intermediate frequencies and persist than their counterparts under weak *directional* selection (which can be modeled using the quantitative genetic framework developed by refs. 6 and 44). However, they also convert additive variance into various forms of interaction variance as they change frequency, and it is unclear to what extent this nonadditive variance might remain correlated with the focal allele across generations, or how the conversion of variance between different statistical partitions impacts linked selection dynamics. This remains an interesting avenue for future work.

Of course, the linked selection effects we study are not the only mechanism through which linkage influences complex trait evolution, particularly under stabilizing selection. By selecting against extreme phenotypes, stabilizing selection generates negative LD between alleles with similar effects, causing a short-term reduction in genetic variance known as the Bulmer effect (46, 47). From the perspective of a single focal site, the Bulmer effect manifests as a negative covariance between the trait-increasing allele and the genetic background. This attenuates the allele’s marginal phenotypic association and, by extension, its marginal fitness effect (i.e., it decreases the site’s “effective” selection coefficient; 48). Although not the primary focus of our study, we found while calibrating our simulations that this reduction in selection strength leads to a long-term increase in *genic* variance that exceeds the short-term reduction caused by negative LD (*SI Appendix*, Fig. S11). Thus, if stabilizing selection is sustained consistently over the timescales on which individual alleles make their transits through the population, then the net impact of linkage may actually be to increase the genetic variance of quantitative traits, rather than to decrease it (*SI Appendix*, Text G), even before accounting for the potential variance increasing effects of BGS that we describe.

### Implications for Natural Populations.

The extent to which background selection shapes genetic architecture in nature depends critically on the underlying model of phenotypic selection and the marginal fitness effects of individual loci. Naturally, for effectively neutral loci, the specific selection model is irrelevant, and the impact of BGS follows standard neutral expectations. And because this is true only for effectively neutral loci, the observation by ref. 16 in *C. elegans* that loci in gene-poor, high-recombination regions explain significantly more gene expression variance implies that if BGS is responsible for this pattern, then much of the genetic variance in question must be effectively neutral. However, the two models predict different effects once selection becomes strong.

Much of the literature on complex trait evolution assumes that stabilizing selection is the norm (49, 50). While this assumption has historically been difficult to assess, several recent analyses in human genetics support the hypothesis, a finding that appears to hold for diseases as well as quantitative traits (21–23, 26, 27, 51). Suppose we accept the maximalist interpretation of this inference and assume that all functional loci contribute to fitness variance via their effects on traits under stabilizing selection. What, then, would be the effect of BGS on functional variation? In humans, the consequences would be straightforward and relatively minor, given the relatively modest local reductions in effective population size. However, in species experiencing stronger reductions, the impact is more significant, as we would expect the contribution to variance to increase across a wide range of sites that would otherwise by strongly selected. While the increase in heterozygosity due to BGS is capped at ≈1.29—and thus can never provide more than a small boost to total genetic variance—the impact on genetic architecture could be substantial, as strongly selected loci, where alleles would typically be confined to low frequencies, would instead have a significant portion of their variance contribution driven by variants drifting to intermediate frequencies. Notably, this dynamic would be expected to operate only to the extent that $\tau <2Ns_{b}$, so that the effects of background selection have time to take effect.

While it seems likely that stabilizing selection plays an important role in shaping much functional variation, it is reasonable to question whether it is the whole picture. It could be that axes of phenotypic integration which experienced a sustained directional mutation–selection balance are simply more difficult to measure, and have thus evaded detection. If a substantial fraction of functional variation is under directional selection, and if most directional selection were derived from traits subject to exponential fitness functions (or some other model in which individual loci have independent fitness effects), then there is no effect on the contribution to variance from strongly selected sites, although the frequency spectra may still be shifted toward large effect loci if $|\gamma |<2Ns_{b}$, though in a less extreme way than under stabilizing selection because of the absence of the slow-down dynamic at intermediate frequencies.

However, models in which selection acts independently predict unreasonably large genetic load (52, 53). This occurs for the same reason that the exponential model predicts large changes in mean fitness due to BGS: If loci fix independently, the accumulation of weakly selected fixations drives mean fitness down by several orders of magnitude—a result inconsistent with the persistence of natural populations. Stabilizing selection offers one resolution to this “load paradox” (53, 54). Strong synergistic epistasis of the kind present in the liability threshold model offers another, for precisely the reason that the threshold model differs from the exponential model in our analysis: the sharp increase in the rate of mean fitness decline with increasing phenotype. (Although we frame our analysis in terms of disease, any model exhibiting this pattern will behave similarly; 21, 52).

Thus, to the extent that individual variants derive their fitness effects from directional mutation–selection balance, there has been a strong theoretical expectation that synergistic epistasis must be involved. Despite this, empirical support for its importance remains mixed (55–61). However, efforts to detect synergistic epistasis are often underpowered because they typically test for interactions between random sets of alleles, when we might expect a priori that it exists only among sets of variants which integrate into the same phenotype, of which there are presumably many (62); but see ref. 63 for a counterexample]. Consequently, it remains difficult to determine how much “evidence of absence” should be inferred from the current scarcity of empirical evidence.

Clarity on the mechanism by which the genetic load paradox is resolved would therefore provide clarity on the scope of background selection’s impact on complex traits. If stabilizing selection is the primary resolution, BGS acts only locally, restricting its variance-reducing effects primarily to small effect loci. If the resolution involves strong synergistic epistasis, as in the threshold model, BGS forces a global compensation that reduces genetic variance uniformly across the effect size distribution, extending its reach to strongly selected loci that are otherwise immune to local effects. The global coupling predicted under synergistic epistasis would be most pronounced in species where the average genome-wide reduction in effective size is large (i.e. species with a high density of deleterious mutations per unit of recombination), or where it varies substantially across different parts of the genome, given that the magnitude of the global compensation is driven more strongly by sites with smaller *B* values. Notably, because this phenomenon manifests through a genome-wide change in the marginal selection coefficients themselves, it would be difficult to detect empirically, other than by demonstrating that both synergistic fitness epistasis and BGS are operating.

## Materials and Methods

### Simulations.

We validated our theoretical predictions using forward-time simulations in SLiM (64). For single-site models, we simulated focal sites evolving under directional or underdominant selection within a recombining chromosome of length 1 Morgan, subject to background selection driven by deleterious mutations at linked sites. For every simulated allele, we recorded its frequency each generation, and used these recorded frequencies to compute the expected lifetime contribution to heterozygosity, and the sojourn time at each frequency. To plot results for the exponential model, we take advantage of the theoretical equivalence between the exponential model and the single site additive model, averaging over the two possible ancestral states for the allele at long term equilibrium. For the liability threshold and stabilizing selection models, we simulated chromosomes with causal sites interspersed among deleterious background sites. In these phenotypic simulations, we measure the genetic variance of the phenotype at regular intervals, with and without background selection. Full details of the simulation parameters and measurement protocols are provided in *SI Appendix*.

### Use of Generative AI Tools.

We used generative AI tools, including Gemini 2.5 Pro and Gemini 3.0 Pro, as well as ChatGPT-5, to help draft and edit text in this manuscript, and to produce and edit code for plotting figures. All text and code drafted or edited in this manner was thoroughly checked and edited further before inclusion in the final manuscript. We take full responsibility for the contents of this manuscript.

## Supplementary Material

Appendix 01 (PDF)

## Data Availability

Scripts to reproduce all analyses are available at https://github.com/xinyli/BGS_msdb.git (65).
