# Supplementary material for: Background selection in recombining genomes and its consequences for the maintenance of variation in complex traits
Source: Proc Natl Acad Sci U S A. 2026 Mar 31;123(14):e2513613123. doi: 10.1073/pnas.2513613123 (PMC13056065; doi:10.1073/pnas.2513613123)
Supplement: Supplementary file 1 — Appendix 01 (PDF) [file pnas.2513613123.sapp.pdf]

Supporting Information for

Background selection in recombining genomes and its consequences for the maintenance of variation in complex traits

Xinyi Li, Jeremy J Berg

Jeremy J Berg.
E-mail: jjberg@uchicago.edu

This PDF file includes:

Supporting text
Figs. S1 to S11
Table S1
SI References

Supporting Information Text

A. Methods

Simulation of single-site selection models. To validate the predictions on diversity reduction by BGS in our single-site models, we performed SLiM simulations (1) on a single site under directional or underdominant selection across a range of selection coefficients. In each case, we simulated $5,000$ recombining diploid genomes, with each genome having $M = 4 \times 10^5$ background sites, and a single focal site in the middle of the chromosome. The per-site recombination rate is set to 2.5×10^{-6}, such that the map length is equal to 1 Morgan. The mutation rate at each of the M background sites is 2.5×10^{-7}. When we simulate without background selection, all mutations at the M background sites other than the focal one are neutral. When we simulate with background selection, all mutations at these M background sites have a selection coefficient $s = -0.005$. With $\nu = 2.5 \times 10^{-7}$ and $r = 2.5 \times 10^{-6}$, this produces a B of 0.82. To vary the background selection strength, we tune the mutation rate (ν) with a fixed recombination rate (r) to produce a specified B ($B = e^{-\lambda} = e^{-2\frac{\nu}{r}}$).

The mutation rate at the focal site is set to zero, and we introduce mutations at this site "manually". We first let the simulation of the background sites run for $2N$ generations so that the population reaches mutation-selection balance. After this burn-in period, we introduced a single selected allele at the focal site in the center of the genome.

To measure the reduction of heterozygosity by BGS, we adopt the method used by (2) to measure the expected total contribution to heterozygosity of a new mutation during its transit through the population (H_{tot}). This is given by the sum of heterozygosity at the focal site during the T generations that the mutation segregated in the population,

$$H_{tot} = 2\sum_{i=1}^{T} x_i(1 - x_i), \qquad \text{[A.1]}$$

which has a theoretical expectation of $\mathbb{E}[H_{tot}|\gamma] = h_d(\gamma)$, or $\mathbb{E}[H_{tot}|\tau] = h_u(\tau)$, depending on the model, and is thus proportional to the expected heterozygosity for a single site at a random sampling time in the low mutation limit. For each selection coefficient, we average the value of H_{tot} across 10^7 separate focal sites. These mutations were simulated across 10^4 replicate burn-ins, with 10^3 mutations considered per burn-in. We then take the ratio of H_{tot} under BGS relative to no BGS as our estimator of the impact of background selection on the expected heterozygosity. To obtain a standard error, we treat the value of H_{tot} for a single mutation's transit through the population as a single data point, and bootstrap across these values.

To construct the site frequency spectrum (SFS), we record the allele frequency of each new mutation at every generation during its sojourn in the population. To validate the frequency-dependent B approximation, we generate multiple SFS datasets in which ν and r are held constant, ensuring the same expected reduction in neutral diversity across datasets. We then vary the selection coefficients of background mutations, $s_b \in \{-0.006, -0.01, -0.05\}$, as well as an equal mixture of these three coefficients.

Simulation of complex Traits.

Exponential selection model. To plot the reduction in variance due to background selection under the exponential model, we take advantage of the theoretical equivalence between the exponential model and the single-site additive model. To this end, we simulate a single site under directional selection with a fixed selection coefficient, as described above, except that each time a mutation ends its transit through the population and we revert the simulation back to the end of the burn-in, we choose the sign of the effect of the next mutation to be opposite to that of the allele that fixed at the end of the previous mutation's transit; i.e. if it was the deleterious allele that fixed, then the next mutation would be beneficial, and vice versa. This scheme simulates the long-term fixation dynamics under the exponential model. Calculations regarding the reduction in heterozygosity due to BGS are then otherwise performed as described above in the single-site case.

Threshold selection model. In this case, we simulate under the full threshold model, again with $5,000$ diploid individuals. For each choice of $p_T = \frac{T}{2La}$ in the single-effect-size model, we simulated $L = 10^5$ causal sites which contribute to phenotypic variation, set $a = 1$, and choose T so as to attain the correct value of p_T. We set the mutation rate for causal sites to $\mu = 10^{-7}$. All causal sites are equally spaced along the chromosome. Between each pair of causal sites, there are 25 BGS sites, as well as a single neutral site (these neutral sites are used to empirically check the strength of the BGS effect, i.e. the B value, against the expectation). For most of our simulations, the recombination rate among adjacent sites is set to $r = 2 \times 10^{-7}$, and the mutation rate at BGS sites is set to $\nu = 2 \times 10^{-8}$, to yield an expected B value of $e^{-0.2} \approx 0.82$, approximately equal to the estimated human average. To ensure that all causal sites experience a similar background selection effect, we added 6.5×10^5 negatively selected sites on each end of the chromosomes, such that the chromosome has a total map length of 0.8 Morgan. In the simulations where we vary the strength of the BGS effect, we do so by varying the mutation rate for BGS sites, ν, with the recombination rate held constant.

To compare traits with and without background selection, we first simulate a trait with the environmental variance tuned so that the heritability is $h^2 = 0.5$ in the absence of background selection (i.e. setting $s = 0$ for all BGS sites). We then rerun the same simulation with background selection turned on (i.e. with $s = -0.005$ for all BGS sites), holding the environmental variance constant at the same value used in the no background selection case. In all cases, we set the fitness cost of being across the threshold at $C = 0.2$. An individual's fitness is then computed as a product of their fitness effect due to the trait together with independent multiplicative contributions from the BGS sites.

To compare the single-effect-size trait model to the single-site results, we assign a scaled selection coefficient to each set of trait simulations. This quantity measures the average selection coefficient that causal variants experience in the trait model. For each choice of $p^{+}(a)$ in the simulations, we estimated the unscaled selection coefficient by regressing individual fitness against genotypes in the absence of background selection. This regression was performed for each causal site segregating in a given generation and repeated across multiple generations, with the average slope serving as our estimate. We then obtained the scaled selection coefficient by multiplying this estimate by $2N$. The corresponding simulation run, conducted with the same $p^{+}(a)$ but with background selection included, was assigned the same scaled selection coefficient. In principle, in the small effect regime we could obtain the scaled selection coefficient analytically using Eq. (18), but this method breaks down for large effects, so we use the empirical regression-based estimate across the full range.

Stabilizing Selection. Our simulations under stabilizing selection are similar to those under the threshold model, with a few key differences. Notably, to avoid the Bulmer effect in our simulations of stabilizing selection (see Supplementary section F and Figure S11), we had to increase the harmonic mean recombination rate between pairs of causal sites (3–6). In these simulations, we set $L = 5.4 \times 10^4$, and split the causal sites across 6 independent chromosomes, so that each chromosome has 9000 causal sites. Between each pair of adjacent causal sites on a given chromosome, there are 98 BGS sites and 1 neutral site. The recombination rate is set to $r = 10^{-6}$ per base pair, while the mutation rate is $\mu = \nu = 10^{-7}$ for both causal and BGS sites. We set the width of the stabilizing selection surface $\omega^2 = 2N$ and vary the effect size for each site a.

To compare the single-effect-size trait model to the single-site results, for each choice of a, we measure the average heterozygosity of all the causal sites in the simulations without background selection over multiple generations and solved for the τ with Eq. (23). We then multiply this estimate by $2N$ and assign it to the simulations with the same a with and without background selection.

B. The frequency spectrum under background selection in a recombining background

Table S1. Notation table for Supplementary Text B

Symbol	Description
**Model Parameters**	
N	Diploid population size.
M	Total chromosome length.
r	Per-site recombination rate.
ν	Per-site deleterious mutation rate.
s_b	Selection coefficient against a single background deleterious allele.
s	Direct selection coefficient of the focal allele.
γ	Population-scaled direct selection coefficient (e.g., $2Ns$).
$g_b(s_b)$	Distribution of fitness effects (DFE) for background mutations.
t	Time in generations.
**Frequency Spectrum & Allele Notation**	
q	Allele frequency.
$q(t)$	Expected allele frequency at time t.
θ	Population-scaled neutral mutation rate (i.e., $4N\mu$).
$\xi(q)$	Standard neutral frequency spectrum (proportional to θ/q).
$\xi(q \mid \gamma)$	Frequency spectrum for a directly selected allele with coefficient γ.
$\pi(p \mid \gamma)$	Fixation probability of a selected allele at frequency p.
**Classic BGS Model (Pre-existing Mutations)**	
T_{s_b}	Purging timescale for a haplotype with one deleterious allele ($\approx 1/s_b$).
M_{s_b}	Characteristic block length for classic BGS ($2s_b/r$).
λ	Expected number of **pre-existing** mutations on a block of length M_{bgs} ($2\nu/r$).
B_i	Proportion of haplotypes carrying i deleterious alleles in an M_{s_b} block.
B_0	Proportion of haplotypes free of deleterious alleles ($e^{-\lambda}$).
$\xi_{B_0}(q)$	Classic BGS approximation for the frequency spectrum ($\approx B_0\xi(q)$).
$q(T_{s_b})$	Frequency threshold for the classic BGS approximation ($\approx 1/(2Ns_bB_0)$).
$B(q)$	Frequency spectrum skew in weak mutation limit ($e^{-\lambda(1-e^{-4Ns_bq})}$).
$\xi_B(q \mid \gamma)$	Frequency-dependent BGS approximation for a directly selected allele.
**Fitness Drag Model (New Mutations)**	
M_t	Expected length of the unrecombined block at time t ($2/(rt)$).
D_t	Expected number of new mutations accumulated on a block of length M_t (also $= \lambda$).

Continued on next page

Table S1 – *Continued from previous page*

Symbol	Description
s_{drag}	Effective selection coefficient due to new mutations (λs_b).
T_{drag}	Purging timescale due to fitness drag ($\approx 1/s_{drag} = 1/(\lambda s_b)$).
M_{drag}	Characteristic block length for the fitness drag process ($2s_{drag}/r = 2\lambda s_b/r$).
$q(T_{drag})$	Frequency threshold for the fitness drag model ($1/(2Ns_e)$).
**Fitness Variance & (7) Comparison**	
σ_W	Standard deviation of fitness.
$\sigma_W(t)$	SD of fitness for equilibrium blocks of length M_t ($\sqrt{M_t s_b \nu}$).
$\sigma_{W,M_{drag}}$	SD of fitness for equilibrium blocks of length M_{drag} (also $= s_{drag}$).
U_D	Total deleterious mutation rate per block (in the non-recombining model).
$T_{decline}$	Purging timescale in the non-recombining model ($\approx 1/(s_b\sqrt{\lambda})$).
$q(T_{decline})$	Frequency threshold in the non-recombining model ($\approx 1/(2Ns_b\sqrt{\lambda})$).

Classic background selection: removal due to pre-existing mutations. We consider a chromosome of length M that recombines at a rate r per site, on which deleterious alleles with selection coefficient s_b arise at a rate ν per site. We are interested in the fate and frequency distribution of a focal allele that arises on a random chromosome in this population at time $t = 0$.

After t generations have passed, the distance (in each direction) from the focal allele to the first recombination event is exponentially distributed with rate rt. The length of the block that remains associated with the focal mutation without any intervening recombination is on expectation

$$M_t = \frac{2}{rt} \tag{B.1}$$

(the factor of 2 comes from the fact that there is an uninterrupted block with expected length $^1/_{rt}$ in each direction). A haplotype carrying a single deleterious allele is purged from the population in

$$T_{s_b} \approx \frac{1}{s_b} \tag{B.2}$$

generations on expectation, so the expected length of a haplotype block over which selection discriminates between focal alleles linked to one vs. zero deleterious alleles is

$$M_{s_b} = \frac{2}{rT_{s_b}} = 2\frac{s_b}{r}. \tag{B.3}$$

This defines a characteristic length scale in the classic model of background selection in a recombining background. At mutation-selection balance, the expected number of deleterious alleles in a window of this size is

$$\lambda = M_{s_b}\frac{\nu}{s_b} = 2\frac{\nu}{r}, \tag{B.4}$$

and the distribution is approximately Poisson, with the proportion of haplotypes carrying i deleterious alleles within this window given by

$$B_i = \frac{\lambda^i}{i!}e^{-\lambda}. \tag{B.5}$$

This result is the source of standard "reduced effective population size" approximation for a long recombining chromosome. In this approximation, only a neutral allele that arises on a length M_{bgs} haplotype free of deleterious mutations (which occurs with probability $B_0 = e^{-\lambda}$) has any hope of being seen with an appreciable frequency in the population. Therefore, while the neutral frequency spectrum in the absence of background selection would be expected to take the form

$$\xi(q) = \frac{\theta}{q}, \tag{B.6}$$

the classic background selection approximation implies a systematic reduction by a factor of B_0 in the number of alleles reaching any given frequency

$$\xi_{B_0}(q) \approx B_0\xi(q). \tag{B.7}$$

Because the effect of selection is not instantaneous, this approximation is only valid above a certain critical frequency. To determine what this frequency is, consider the transit of a focal neutral allele linked to a haplotype block carrying a single deleterious allele. This haplotype block originated within the population of mutation free blocks, and all other blocks carrying at least one mutation will soon be extinct, so we can think of it as evolving within a population of reduced effective size NB_0.

As we discussed above, the allele will be eliminated by selection on a timescale of $T_{s_b} = {}^1\!/_{s_b}$ generations, and so will be prohibited from reaching frequencies that cannot be reached via genetic drift in that amount of time. For small values of t, the expected frequency of a neutral allele in a diploid population of size NB_0 conditional on not yet having been lost is approximately $q\left(t\right) \approx {}^{t}\!/_{2NB_0}$. Therefore, haplotypes carrying at least one deleterious allele are limited to frequencies no greater than approximately

$$q\left(T_{s_b}\right) = \frac{T_{s_b}}{2NB_0} = \frac{1}{2Ns_bB_0}. \tag{B.8}$$

Eq. (B.8) thus gives an approximate threshold for the frequency at which Eq. (B.7) becomes valid. This is the threshold suggested by (7), provided that one replaces $\lambda = \frac{U_D}{s}$ in their haploid model of a non-recombining block with $\lambda = 2\frac{\nu}{r}$ in our diploid model of a recombining chromosome.

Length- and time-scale of removal due to selective drag of new mutations. The classic background selection model considered above assumes that the most relevant process is the removal of the haplotype that the focal allele arises on, due to selection against mutations that are already present there when the allele arises. If the deleterious mutation rate is high relative to the recombination rate, then it could also be removed due to selection against deleterious mutations accumulating after it arises. After t generations have passed, the haplotype associated with the deleterious allele has expected length $M_t \approx {}^2\!/_{rt}$ (see eq. Eq. (B.1)), and so on expectation has accumulated

$$D_t = M_t\nu t = 2\frac{\nu}{r} = \lambda \tag{B.9}$$

additional deleterious alleles since it arose. It is notable that this expectation is independent of time, as the deleterious alleles that are separated by recombination are replaced by new deleterious mutations arising on the (on expectation, slightly shortened) block that remains associated. While their expectations are numerically equal ($D_t = \lambda$), it is important to distinguish these two quantities conceptually: D_t is the expected number of new mutations accumulated on a shrinking, age-t block, whereas λ is the expected number of pre-existing mutations found on an equilibrium block of length M_{bgs}.

This time-independence represents a key distinction from the non-recombining model of (7), in which the absence of recombination means that the number of new mutations accumulates linearly with time (i.e. $D_t \propto t$). In that case, the process is one of steady fitness decline as the haplotype becomes progressively loaded with mutations. In the recombining model, the balance between recombination and mutation establishes a constant, time-independent expected mutational load, $D_t = \lambda$, for the block associated with the focal allele. (We say more about the similarities and distinctions between the two models below.)

Each recombination event generates a new set of associations with deleterious alleles at distances greater than the expected ${}^1\!/_{rt}$ distance to the edge of the block. However, these new associations are samples from the equilibrium distribution, and therefore have contributions that are on average equal to the population mean, and thus have no net effect on the mean fitness of the focal block. The result is that in the early generations before selection has had a chance to shape the trajectory of our focal allele, its expected fitness drag due to new mutations is constant:

$$s_{drag} = D_t s_b = \lambda s_b. \tag{B.10}$$

This suggests that a typical allele at the focal sites should be removed due to this immediately accumulated selective drag on a timescale of

$$T_{drag} = \frac{1}{s_{drag}} = \frac{1}{\lambda s_b} \tag{B.11}$$

generations, and that the expected length of the haplotype block that is removed with it is

$$M_{drag} = \frac{2}{rT_{drag}} = 2\frac{s_{drag}}{r} = 2\frac{\lambda s_b}{r} = \lambda M_{s_b}. \tag{B.12}$$

This process of selection against newly arising mutations on the background of the focal allele will be important for frequencies less than

$$q\left(T_{drag}\right) = \frac{T_{drag}}{2N} = \frac{1}{2Ns_{drag}} = \frac{1}{2N\lambda s_b}, \tag{B.13}$$

but becomes unimportant above this threshold because any alleles exceeding this frequency are likely outliers that have accumulated fewer deleterious mutations than average.

As we indicated above, this process is only important to the dynamics if recombination is weak relative to mutation, in which case the timescale of removal due to the accumulation of deleterious mutations is faster than the timescale on which mutation-free haplotypes are preserved, i.e. $T_{drag} \ll T_{s_b}$. Intuitively, this condition translates into a requirement that $\lambda \gg 1$.

More precisely, we might think that in order for the effects of the immediate fitness drag to be important, the threshold frequency at which this fitness drag has substantially reshaped the frequency spectrum must be below the threshold frequency at which the classic background approximation begins to apply, i.e. $q\left(T_{drag}\right) \ll q\left(T_{s_b}\right)$. This is equivalent to the condition $B_0T_{drag} \ll T_{s_b}$. This leads to the more restrictive condition $\lambda \gg e^{-\lambda}$ on the value of λ at which the fitness drag becomes important. This is solved by $\lambda \gg W_0\left(1\right) \approx {}^1\!/_2$, i.e. $\nu \gg {}^{rW_0(1)}\!/_2 \approx {}^r\!/_4$, where W_0 is the main branch of the Lambert W function and, very roughly, $W_0(1) \approx {}^1\!/_2$. This condition more closely matches our observations from simulations that the fitness drag begins to impact the frequency spectrum roughly when $\lambda > {}^1\!/_2$ (Figure S1).

Comparison to (7) non-recombining model This "fitness drag" process provides a close parallel to the "fitness decline" process in the non-recombining model of (7). In that model, λ is defined as $\lambda = {}^{U_D}/_{s_b}$, where U_D is the total deleterious mutation rate for the entire non-recombining block. For an equivalent value of λ, both models predict the same overall reduction in the effective population size ($B_0 = e^{-\lambda}$). Furthermore, if s_b is also held constant, the upper frequency threshold $q(T_{s_b})$—which defines the frequency below which the classic BGS approximation of Eq. (B.7) breaks down—is also the same. This equivalence follows directly from the validity of the "effectively non-recombining block" approximation, which simply converts the recombining problem into the corresponding non-recombining one for alleles above the relevant threshold.

The most significant difference between the two models lies in their predictions regarding the impact of selection on alleles at very low frequencies. Notably, because the "block length" is fixed in the non-recombining model, there is no contribution from the accumulation of deleterious alleles at distances beyond the characteristic block length. Instead, the block must first decline in fitness by accumulating deleterious mutations. As a result, we speak of a process of "fitness decline" and its consequences, rather than the "fitness drag" process described above for the recombining model.

In the non-recombining model, these accumulated mutations become important once the fitness of the block has declined by an amount equal to the equilibrium standard deviation in fitness, $\sigma_W = s_b\sqrt{\lambda}$. At this point, the fitness deficit relative to the rest of the population is substantial enough that selection can effectively remove the allele.

Ignoring the impact of selection on short timescales, fitness declines linearly with time in the non-recombining model (i.e., $s_e(t) \approx (U_D t)s_b$). Setting the accumulated load $(U_D t)s_b$ equal to the equilibrium standard deviation $\sigma_W = s_b\sqrt{\lambda}$ and solving for t, the timescale on which an allele declines sufficiently in fitness to be removed by selection is

$$T_{decline} \approx \frac{s_b\sqrt{\lambda}}{U_D s_b} = \frac{1}{s_b\sqrt{\lambda}} = \frac{1}{\sigma_W}. \quad \text{[B.14]}$$

Consequently, in the non-recombining model, we expect that for frequencies below a threshold of

$$q(T_{decline}) \approx \frac{T_{decline}}{2N} = \frac{1}{2Ns_b\sqrt{\lambda}} = \frac{1}{2N\sigma_W}, \quad \text{[B.15]}$$

selection has not yet had an opportunity to act, so the frequency spectrum should be approximately neutral. Once an allele reaches this frequency, however, it is likely to have accumulated a fitness deficit of approximately $s_e\left(T_{decline}\right) = \sigma_W$, and will therefore be eliminated on a timescale of roughly $T_{decline} = {}^1/_{\sigma_W}$ additional generations. During this time, the allele's frequency can increase by at most another factor of roughly 2. Thus, Eq. (B.15) provides the correct order-of-magnitude limit on the frequency at which the accumulation of new mutations dominates the dynamics.

Notably, the equivalent threshold in the recombining model, $q\left(T_{drag}\right) = {}^1/_{2Ns_b\lambda}$ (Eq. (B.13)), is lower by a factor of ${}^1/\sqrt{\lambda}$. This difference reflects the difference in dynamics between the two models which can also be understood in terms of an appropriate standard deviation of fitness. Specifically, in the recombining model, the drag s_{drag} is equal to the equilibrium fitness standard deviation of blocks with length M_{drag} (i.e., blocks of the characteristic length eliminated by this process). The equilibrium distribution of pre-existing mutations on a block of length M_{drag} is Poisson with mean $M_{drag}(\nu/s_b) = (2\lambda s_b/r)(\nu/s_b) = \lambda^2$. The standard deviation of fitness among blocks of this length is therefore:

$$\sigma_{W,M_{drag}} = \sqrt{\lambda^2 s_b^2} = \lambda s_b = s_{drag}. \quad \text{[B.16]}$$

This equality provides a dynamic interpretation of the removal timescale, T_{drag}. When an allele is young ($t \ll T_{drag}$), its associated block is long ($M_t \gg M_{drag}$). The background standard deviation of fitness for very young blocks is consequently very large ($\sigma_W(t) = \sqrt{M_t s_b \nu} \gg \sigma_{W,M_{drag}}$), and the allele's constant fitness drag is small relative to this background variation. As the allele ages and t approaches T_{drag}, its associated block shrinks toward length M_{drag}, and the background fitness variance shrinks with it. When the block reaches length M_{drag}, the background variance shrinks to equal the fitness drag ($\sigma_{W,M_{drag}} = s_{drag}$), and it is around this time that the allele is likely to be purged.

This difference in mechanism—the accumulation of fitness deficit versus a constant fitness drag—arises because, in the recombining model, deleterious mutations far beyond the characteristic block length of classic BGS exert an immediate influence. This extended range accelerates the onset of strong selection: the average fitness deficit becomes visible to selection on a timescale shorter by a factor of

$$\frac{T_{drag}}{T_{decline}} = \frac{1}{\sqrt{\lambda}} \quad \text{[B.17]}$$

compared to the equivalent non-recombining model, and consequently that BGS should skew the frequency spectrum at frequencies that are also lower by a factor of

$$\frac{q\left(T_{drag}\right)}{q\left(T_{decline}\right)} = \frac{1}{\sqrt{\lambda}} \quad \text{[B.18]}$$

This perspective on the recombining model is somewhat at odds with the idea that the allele is under strong, constant selection immediately. For early times $t \ll T_{drag}$, when the drag is much smaller than the background variance ($s_{drag} \ll \sigma_W(t)$), the argument made by (7) in the context of the non-recombining model would suggest that the allele should behave neutrally at first due to the noise of the background. This tension is in some part real, and reflects the fact that all of these are coarse

approximations for an extremely complex process. However, this tension is partially resolved by the fact that the argument from (7) is a bit too strong, and also partially by the difference in dynamics between the two models.

In the non-recombining model, the fitness deficit grows linearly relative to a fixed standard deviation, meaning the ratio also scales linearly with time:

$$\frac{s_e(t)}{\sigma_W} \approx \frac{(U_D t)s_b}{s_b\sqrt{\lambda}} = ts_b\sqrt{\lambda} = \frac{t}{T_{decline}}. \quad \text{[B.19]}$$

Consequently, the time-averaged value of $^{s_e(t)}/_{\sigma_W}$ over the interval $[0, T_{decline}]$—the period during which the allele's fitness deficit increases from 0 to σ_W—is $\int_0^{T_{decline}} {}^{t}/_{T_{decline}} \mathrm{d}t = {}^1/_2$. This suggests that if one wishes to approximate the early dynamics using a constant selection coefficient, $^{\sigma_W}/_2$ may be a more useful choice than 0.

In contrast, in the recombining model, the background fitness standard deviation shrinks with $^1/_{\sqrt{t}}$, while the fitness deficit remains constant. Consequently, the ratio scales with the square root of time:

$$\frac{s_{drag}}{\sigma_W(t)} \approx \frac{\lambda s_b}{\sqrt{s_b\lambda/t}} = \sqrt{ts_b\lambda} = \sqrt{\frac{t}{T_{drag}}}. \quad \text{[B.20]}$$

The time-average of $^{s_{drag}}/_{\sigma_W(t)}$ over the interval $[0, T_{drag}]$ is therefore $\int_0^{T_{drag}} \sqrt{^{t}/_{T_{drag}}}\mathrm{d}t = {}^2/_3$. This reflects the fact that the ratio of the fitness deficit to the background standard deviation is higher on average during the early phases of the allele's trajectory than in the non-recombining case (Figure S5). This suggests that to approximate the low-frequency dynamics of the recombining model using a single constant selection coefficient, one should use $^{2s_{drag}}/_3$, thereby discounting for the impact of background fitness variance in the earliest generations.

These arguments suggest that the frequency spectrum is more strongly distorted in recombining models than in equivalent non-recombining ones, not only because the focal allele becomes "visible" to selection faster, but also because it spends more time near this visibility threshold before reaching it. While these arguments are heuristic, the central conclusion is deeply intuitive: the suppression of the frequency spectrum extends to lower frequencies in large recombining genomes than predicted by non-recombining models with equivalent reductions in effective size, due to the action in early generations of deleterious mutations at recombination distances greater than the characteristic length-scale of the classical recombining BGS model.

The frequency spectrum at low frequencies.

The strong recombination/weak mutation regime.

Neutral alleles The arguments in the preceding sections allow us to understand the shape of the frequency spectrum for neutral alleles at very low and very high frequencies. Roughly, when $\lambda \ll {}^1/_2$ (or equivalently, when $B_0 \gg 0.6$) recombination is too fast relative to mutation for the process of fitness decline to be relevant (i.e. $T_{drag} \gg B_0 T_{s_b}$), and so the frequency spectrum is shaped entirely by the effect of selection acting against deleterious alleles that are already present when the focal neutral allele arises. Based on the approximations given above, this suggests that

$$\xi_{B_0}(q) = \begin{cases} \frac{\theta}{q} & q \ll \frac{1}{2Ns_bB_0} \\ \frac{\theta}{q}B_0 & q \gg \frac{1}{2Ns_bB_0}. \end{cases} \quad \text{[B.21]}$$

We can push this perspective further and obtain an approximation that is valid across all frequencies by taking advantage of the assumption that the focal allele is eliminated by deleterious alleles already present on its background when it arises. To this end, consider that with probability B_i the focal allele arises on a haplotype that carries i deleterious alleles within a distance of $^{M_{bgs}}/_2 = {}^{s_b}/_r$ in either direction. Provided that carrying *any* deleterious alleles on a haplotype of this length is rare in general (consistent with the assumption that $B_0 \gg 0.6$), then standard diffusion results tell us a neutral allele on a haplotype with i deleterious alleles will spend approximately $\frac{2}{q}e^{-4Ns_biq}$ generations at frequency q. We can therefore approximate the frequency spectrum across the full range of frequencies as

$$\xi_B(q) \approx \xi(q)\sum_{i=0}^{\infty} B_i e^{-4Ns_biq} \quad \text{[B.22]}$$

$$= \xi(q)\, e^{-\lambda}\sum_{i=0}^{\infty}\frac{\left(\lambda e^{-4Ns_bq}\right)^i}{i!} \quad \text{[B.23]}$$

$$= \xi(q)\, B(q,\lambda), \quad \text{[B.24]}$$

where

$$B(q,\lambda) = e^{-\lambda}\sum_{i=0}^{\infty}\frac{\left(\lambda e^{-4Ns_bq}\right)^i}{i!} \quad \text{[B.25]}$$

$$= e^{-\lambda\left(1-e^{-4Ns_bq}\right)}. \quad \text{[B.26]}$$

is a frequency dependent B value, the form of which follows from the fact that the infinite sum in Eq. (B.25) is the full Taylor series representation of the function $e^{\lambda e^{-4Ns_b q}}$ expanded around the point $\lambda e^{-4Ns_b q} = 0$. We note that the sum in Equation H3 of (7) is essentially identical to our Eq. (B.22) above, although they evaluate it only to zeroth order, i.e., $\xi_B(q) = \xi(q) + \mathcal{O}(\lambda)$.

A distribution of background fitness effects This frequency dependent B value can easily be generalized for a distribution of fitness effects among the sites generating background selection. Suppose that sites responsible for background selection draw their selection coefficients from some distributions, $g_b(s_b)$, and suppose that the largest selection coefficients sampled from this distribution still conform to the simplifying assumptions $s_b \ll Mr$ and $2Ns_b \gg 1$.

Under these assumptions, the number of linked deleterious mutations is Poisson distributed with mean λ, and the selection coefficient of each mutation is drawn independently from $g_b(s_b)$. Since the effect of each mutation on the sojourn time is multiplicative (multiplying the neutral time by $e^{-4Ns_b q}$), the total frequency dependent B value is the expectation of the product of these effects, yielding:

$$\begin{aligned} B(q, \lambda, g_b) &= \sum_{k=0}^{\infty} B_i \left(\mathbb{E}_{g_b}\left[e^{-4Ns_b q}\right]\right)^k \\ &= e^{-\lambda\left(1-\mathbb{E}_{g_b}\left[e^{-4Ns_b q}\right]\right)}. \end{aligned} \quad \text{[B.27]}$$

where $\mathbb{E}_{g_b}\left[e^{-4Ns_b q}\right] = \int g_b(s_b)\, e^{-4Ns_b q} ds_b$. Note that this derivation appears to neglect the fact that the characteristic length scale depends on the selection coefficient. However, while we might expect this dependency to complicate the strong mutation limit, it is irrelevant in the weak mutation limit. In this regime, a haplotype is typically eliminated by a single deleterious allele; consequently, the effective length scale is determined solely by the mutation responsible for the elimination. The result therefore correctly averages over the relevant distribution of length scales.

Directly selected alleles When the focal allele undergoing background selection is itself directly selected, we can find useful approximations by straightforwardly extending the results above under neutrality. First, consider the expected frequency spectrum of a deleterious allele in the absence of linked selection, which is

$$\xi(q \mid \gamma) = 2\frac{e^{2\gamma q}}{q(1-q)}\left(1 - \pi(q \mid \gamma)\right) \quad \text{[B.28]}$$

where

$$\pi(q \mid \gamma) = \begin{cases} q & \gamma = 0 \\ \frac{1-e^{-2\gamma q}}{1-e^{-2\gamma}} & \gamma \neq 0 \end{cases} \quad \text{[B.29]}$$

is the fixation probability of an allele at frequency q with a scaled selection coefficient γ.

In (8)'s classical approximation for weakly selected alleles (i.e. $\gamma \sim 1$), we simply ignore all variants arising on backgrounds other than the mutation free background, i.e.

$$\xi_{B_0}(q \mid \gamma) = B_0 \xi(q \mid \gamma B_0). \quad \text{[B.30]}$$

This approximation extends the "scaled mutation rate" effect of background selection by including a "scaled selection coefficient" effect. The argument for this approximation relies on a separation of timescales between background selection (which acts quickly), and direct selection (which is assumed to act slowly). Alleles reaching frequencies greater than $1/2Ns_bB_0$ must have originated on backgrounds with $i = 0$ deleterious alleles. Once these alleles reach appreciable frequency, new deleterious mutations occurring on their background spread the focal allele across all fitness classes, but because backgrounds with $i > 0$ deleterious alleles are always quickly removed, only the population of size NB_0 is relevant to the evolution of the focal allele. As a result, the balance between drift and selection over the longer timescale on which (weak) direct selection acts is captured by a rescaled version of the population scaled selection coefficient: γB_0.

Combining this classic separation of timescales argument with the frequency dependent B value argument we articulated above, we can approximate the full frequency spectrum of directly selected alleles under weak selection as

$$\xi_B(q \mid \gamma) = \xi(q \mid \gamma B_0) \sum_{i=0}^{\infty} B_i e^{-4Ns_b iq} \quad \text{[B.31]}$$

$$= B(q, \lambda)\xi(q \mid \gamma B_0). \quad \text{[B.32]}$$

Because $B_0 = e^{-\lambda}$ regardless of the value of s_b (provided that $s_b \ll Mr$), this approximation can be straightforwardly extended to the case with a distribution of background fitness effects using Eq. (B.27).

Alternatively, when the strength of direct selection is similar to that of background selection, this separation of timescales does not apply. Direct and background selection act on the same timescale, leading to:

$$\xi_B(q \mid \gamma) = \sum_{i=0}^{\infty} B_i \xi(q \mid \gamma - 2Ns_b i) \quad \text{[B.33]}$$

$$= \xi(q \mid \gamma) \sum_{i=0}^{\infty} B_i e^{-4Ns_b iq} \quad \text{[B.34]}$$

$$= B(q, \lambda)\xi(q \mid \gamma) \quad \text{[B.35]}$$

The strong mutation/weak recombination regime. When recombination is weak relative to mutation (i.e., $\lambda \gg 1/2$), the frequency spectrum is shaped differently. As established, this condition implies $T_{drag} \ll T_{s_b}$, creating two distinct selective thresholds, $q(T_{drag}) \ll q(T_{s_b})$, which define three frequency regimes. In the lowest frequency regime, $q \ll q(T_{drag})$, the frequency spectrum is shaped by the emergence of the time-independent, constant expected fitness drag against the shrinking background fitness variation. In the highest frequency regime, $q \gg q(T_{s_b})$, all alleles originated on the fittest background, and the frequency spectrum is well approximated by the classic rescaled effective population size. For the intermediate regime, where $q(T_{drag}) \ll q \ll q(T_{s_b})$, (7) provides an approximation in the non-recombining model for the form of the frequency spectrum. Here, for completeness, we use heuristic arguments to recapitulate their results in the context of the recombining model.

The key to this regime is that our constant s_{drag} approximation, while useful for describing the behavior at frequencies below the lower threshold $q(T_{drag})$, breaks down at frequencies above it. Specifically, $s_{drag} = \lambda s_b$ represents the expected fitness drag for an allele that has not yet persisted long enough for selection to significantly impact its trajectory. For an allele to reach a frequency $q \gg q(T_{drag})$, it must have been lucky, arising on a background with $i \ll \lambda$ deleterious alleles.

Consequently, the dynamics of the intermediate regime are governed by the "classic" BGS process. While this is the same process as in the $\lambda \ll 1$ case, the required mathematical approximation is different. When $\lambda \ll 1$, the variance of the distribution of i is low, and the fittest class (i.e., $i = 0$) is essentially the same as the population mean. In contrast, when $\lambda \gg 1$, the variance is high, and the lucky alleles that survive into the intermediate regime are extreme outliers, far fitter than the population mean (i.e., $i \ll \lambda$).

When $\lambda \gg 1$, the probability of arising on a background with i deleterious alleles declines quickly as $i \to 0$. It follows that the set of backgrounds reaching frequency q will be dominated by the least fit backgrounds (i.e., those with the highest value of i) that are nonetheless capable of reaching frequency q. We can heuristically derive this least fit class, $i(q)$, via an extreme value argument. To reach a frequency q, an allele must arise on one of the fittest "available" backgrounds. We can estimate the number of "available" backgrounds as $2Ns_b q$ (the number of individuals at that frequency, $2Nq$, divided by the selection timescale, $T_{s_b} \approx 1/s_b$). The fittest available background class, i, is the one that solves the extreme value condition:

$$(2Ns_b q) \cdot B_i \approx 1 \quad \text{[B.36]}$$

where $B_i = \frac{\lambda^i}{i!} e^{-\lambda}$ is the probability of arising on a background with i deleterious alleles within the characteristic block of length M_{bgs} (Eq. (B.5)). Taking the log of both sides and applying Stirling's approximation ($\ln(i!) \approx i \ln i - i$) gives:

$$\ln(2Ns_b q) + \ln(B_i) \approx 0 \quad \text{[B.37]}$$

$$\ln(2Ns_b q) + i \ln \lambda - (i \ln i - i) - \lambda \approx 0 \quad \text{[B.38]}$$

$$\ln(2Ns_b q) \approx \lambda - i - i \ln \lambda + i \ln i \quad \text{[B.39]}$$

$$\ln(2Ns_b q) \approx \lambda - i(1 + \ln \lambda - \ln i). \quad \text{[B.40]}$$

Assuming that the $\ln \lambda$ term dominates, we can approximate $1 + \ln \lambda - \ln i \approx \ln \lambda$ (this holds when λ is large and i is effectively intermediate—i.e., $1 \ll i \ll \lambda$). This allows us to solve for i as a function of q:

$$i(q) \approx \frac{\lambda - \ln(2Ns_b q)}{\ln \lambda} = \log_\lambda \left(\frac{1}{2NB_0 s_b q} \right) \quad \text{[B.41]}$$

$$= \log_\lambda \left(\frac{q(T_{s_b})}{q} \right) \quad \text{[B.42]}$$

This $i(q)$ represents a "self-consistency" condition (equivalent to $k_c(f) + 1$ in (7)), which shows that the dominant fitness class among backgrounds reaching frequency q declines logarithmically with increasing frequency. The alternative form for $i(q)$ in Eq. (B.42) follows from the definition $q(T_{s_b}) = 1/2NB_0 s_b$. This form emphasizes that as q increases, the allele must have been increasingly lucky to survive: to reach higher frequencies, it must have arisen on a background with fewer deleterious alleles, until eventually, to reach frequency $q \approx q(T_{s_b})$, the allele must have arisen on a background with $i = 0$ (by which point this approximation has broken down).

The expected number of generations an allele spends at frequency $q(T_{drag}) \ll q \ll q(T_{s_b})$ is thus approximately

$$\xi_B(q \mid q(T_{drag}) \ll q \ll q(T_{s_b})) \approx B_{i(q)} \cdot \xi_B(q \mid i = i(q), q(T_{drag}) \ll q \ll q(T_{s_b})). \quad \text{[B.43]}$$

We already assumed above that $B_{i(q)} \approx {}^{1}/_{2Ns_bq}$ (Eq. (B.36)). Given that the allele arises in this class, the amount of time spent at frequency q has the approximate form

$$\xi_B(q \mid i = i(q), q(T_{drag}) \ll q \ll q(T_{s_b})) \approx \xi(q)F_{i(q)} \quad \text{[B.44]}$$

where $\xi(q) = {}^{\theta}/_{q}$ is the standard neutral spectrum and $F_{i(q)}$ is a factor that accounts for the suppression of this neutral frequency spectrum by selection in the time since the allele arose. Given the fundamental timescale, $T_{s_b} = {}^{1}/_{s_b}$, of the background selection process, $F_{i(q)}$ can be approximated as

$$F_{i(q)} \approx \frac{1}{\sum_{t=0}^{T_{s_b}} s_{eff}(t)}$$

where $s_{eff}(t)$ is the effective selection coefficient experienced by the allele in generation t. Notably, $s_{eff}(0) = s_b i(q)$, but the allele will experience a range of selective environments over its transit through the population, due to a combination of mutation and recombination events shuffling it across the distribution of fitness backgrounds. This process is complicated, so we might naturally want to make an approximation $\sum_{t=0}^{T_{s_b}} s_{eff}(t) \approx T_{s_b} s_{eff,i(q)}$, for some appropriate average effective selection coefficient, $s_{eff,i(q)}$. Most backgrounds are less fit than the $i(q)$ background on which it arose, which suggests that $s_{eff,i(q)} > s_{eff}(0)$. Alternatively, in order to be found at frequency q, the allele cannot have spent too much time on much less fit backgrounds, or it would have been purged. When one conducts a rigorous analysis of the dynamics (as (7) does for the non-recombining case), one finds that $s_{eff,i(q)} = s_b\sqrt{\lambda i\,(q)}$, which is the geometric mean of the allele's initial selection coefficient $s_{eff}(0) = s_b i(q)$, and the one that it would experience on average if it were sampling the distribution completely at random, i.e., $s_b\lambda$, and therefore nicely satisfies both of these criteria.

It follows that

$$\begin{aligned} F_{i(q)} &\approx \frac{1}{T_{s_b} s_{eff,i(q)}} \\ &= \frac{s_b}{s_{eff,i(q)}} \\ &= \frac{1}{\sqrt{\lambda i\,(q)}}. \end{aligned} \quad \text{[B.45]}$$

Thus, putting together Eq. (B.41)-Eq. (B.45), we have

$$\xi_B(q \mid q(T_{drag}) \ll q \ll q(T_{s_b})) \approx \xi(q) \frac{1}{2Ns_bq\sqrt{\lambda \log_\lambda\left(\frac{1}{2NB_0s_bq}\right)}}. \quad \text{[B.46]}$$

Combining this with the heuristic $s_{eff} = \frac{2}{3}s_{drag} = \frac{2}{3}\lambda s_b$ approximation we derived above for the $q \ll q(T_{drag})$ regime, this allows us to write an approximation for the full spectrum for neutral alleles in this regime as a three-part piecewise function, analogous to Equation I21 in (7):

$$\xi_B(q) \approx \xi\,(q) \begin{cases} e^{-\frac{8}{3}N\lambda s_b q} & q \ll q(T_{drag}) \\ \frac{1}{2Ns_bq\sqrt{\lambda \log_\lambda\left(\frac{1}{2NB_0s_bq}\right)}} & q(T_{drag}) \ll q \ll q(T_{s_b}) \\ B_0 & q \gg q(T_{s_b}). \end{cases} \quad \text{[B.47]}$$

Notably, this approximation neglects the deterministic sweep-like behavior described by (7) at very high frequencies. We expect that an effectively non-recombining block approximation could be derived for this regime as well.

C. Derivations Supporting the Exponential Fitness Model

Selection coefficient. To begin, we divide an individual's phenotype into a contribution from the focal site ℓ, which is $a_\ell g_\ell$ (where $g_\ell \in \{0, 1, 2\}$ is the genotype at site ℓ), and the contribution from all other sites, which we write as $Z_{-\ell}$. Writing $f\,(Z_{-\ell})$ for the density on this background contribution, the expected fitness of an individual with genotype g_ℓ is exactly

$$\mathbb{E}\,[W \mid g_\ell] = \int e^{-\frac{a_\ell g_\ell + Z_{-\ell}}{\eta}} f\,(Z_{-\ell})\, dZ_{-\ell} \quad \text{[C.1]}$$

$$= e^{-\frac{a_\ell g_\ell}{\eta}} \int e^{-\frac{Z_{-\ell}}{\eta}} f\,(Z_{-\ell})\, dZ_{-\ell} \quad \text{[C.2]}$$

The ability to factor the fitness effect of the focal site ℓ out of the total effect is a special property of the exponential model, which allows us to rescale the relative fitnesses so that independent of any details about the background, $Z_{-\ell}$, we can write

$$\mathbb{E}\,[W \mid g_\ell] = e^{-\frac{a_\ell g_\ell}{\eta}}, \quad \text{[C.3]}$$

228 without any loss of generality or approximation.

The expected change in frequency for an allele with effect a and frequency x can be written as

$$\mathbb{E}\left[\Delta x \mid a, x, \eta\right] = s(a,x)x(1-x) \tag{C.4}$$

where

$$s(a,x) = \frac{1}{2}\frac{1}{\overline{W}}\frac{d\overline{W}}{dx}. \tag{C.5}$$

is the marginal effect of the allele on fitness. The mean fitness is equal to

$$\overline{W} = (1-x)^2 \cdot 1 + 2x(1-x)e^{-\frac{a}{\eta}} + x^2 e^{-\frac{2a}{\eta}} \tag{C.6}$$

$$= \left(1 - x + xe^{-\frac{a}{\eta}}\right)^2 \tag{C.7}$$

so that

$$\frac{d\bar{W}}{dx} = 2\left[(1-x) + xe^{-\frac{a}{\eta}}\right]\left(e^{-\frac{a}{\eta}} - 1\right) \tag{C.8}$$

and therefore

$$s(a,x) = \frac{1}{\left[(1-x) + xe^{-\frac{a}{\eta}}\right]^2} \cdot \left[(1-x) + xe^{-\frac{a}{\eta}}\right]\left(e^{-\frac{a}{\eta}} - 1\right) \tag{C.9}$$

$$= \frac{e^{-\frac{a}{\eta}} - 1}{(1-x) + xe^{-\frac{a}{\eta}}}. \tag{C.10}$$

Eq. (C.10) is still exact. Assuming that $a \ll \eta$, $xe^{-\frac{a}{\eta}} \approx x$, and $e^{-\frac{a}{\eta}} - 1 \approx -\frac{a}{\eta}$, so we can make the approximation

$$s(a,x) \approx -\frac{a}{\eta}. \tag{C.11}$$

Impact of BGS on the Mean Phenotype and Fitness Load. Here we derive the effect of background selection on the mean phenotype and the corresponding fitness load. The mean phenotype is given by the sum of contributions from sites fixed for the trait-increasing allele. At mutation-selection-drift balance, the fraction of sites fixed for the trait-increasing (deleterious) allele with scaled selection coefficient γ is

$$p^+(\gamma) = \frac{1}{1 + e^{2\gamma}}. \tag{C.12}$$

In the presence of background selection, the effective population size is reduced by a factor B, rescaling the selection coefficient to γB. The change in the fraction of fixed deleterious alleles is therefore

$$\Delta p^+(\gamma B) = p^+(\gamma B) - p^+(\gamma) = \frac{1}{1 + e^{2\gamma B}} - \frac{1}{1 + e^{2\gamma}}. \tag{C.13}$$

The shift in the mean phenotype is the sum of the effects of these additional fixed alleles across all L loci:

$$\bar{G}_B - \bar{G} = 2La\left(p^+(\gamma B) - p^+(\gamma)\right). \tag{C.14}$$

To compare this shift to the scale of the standing genetic variation, we normalize by the genetic standard deviation $\sqrt{V_G} = \sqrt{\frac{\theta}{\gamma}b(\gamma)}$, where $b(\gamma) = \tanh(\gamma)$:

$$\frac{\bar{G}_B - \bar{G}}{\sqrt{V_G}} = 2La\left(p^+(\gamma B) - p^+(\gamma)\right)\sqrt{\frac{\gamma}{\theta b(\gamma)}} \tag{C.15}$$

$$= \gamma\frac{L}{\sqrt{\theta}}\sqrt{\frac{b(\gamma)}{\gamma}}\frac{2\left(p^+(\gamma B) - p^+(\gamma)\right)}{b(\gamma)}. \tag{C.16}$$

For weakly selected sites ($\gamma \ll 1$), we can approximate $p^+(\gamma) \approx \frac{1}{2}(1-\gamma)$ and $b(\gamma) \approx \gamma$. Substituting these approximations yields:

$$\frac{\bar{G}_B - \bar{G}}{\sqrt{V_G}} \approx \gamma\frac{L}{\sqrt{\theta}}\frac{2\left[\frac{1}{2}(1-\gamma B) - \frac{1}{2}(1-\gamma)\right]}{\gamma} = \gamma(1-B)\frac{L}{\sqrt{\theta}}. \tag{C.17}$$

229 Since $L \gg \sqrt{\theta}$, this standardized shift is substantial ($\gg 1$) even for moderate background selection.

This shift in the mean phenotype results in a decline in mean fitness. The ratio of mean fitness with BGS to mean fitness without BGS is:

$$\frac{W(\bar{G}_B)}{W(\bar{G})} = \frac{e^{-\bar{G}_B/\eta}}{e^{-\bar{G}/\eta}} = e^{-\frac{1}{\eta}(\bar{G}_B - \bar{G})}. \tag{C.18}$$

Substituting the expression for the shift in the mean:

$$\frac{W(\bar{G}_B)}{W(\bar{G})} = \exp\left[-\frac{2La}{\eta}\left(p^+(\gamma B) - p^+(\gamma)\right)\right]. \tag{C.19}$$

Using the relation $\frac{2Na}{\eta} \approx \gamma$ (so $\frac{a}{\eta} \approx \frac{\gamma}{2N}$), this becomes:

$$\frac{W(\bar{G}_B)}{W(\bar{G})} = \exp\left[-\frac{L\gamma}{N}\left(p^+(\gamma B) - p^+(\gamma)\right)\right]. \tag{C.20}$$

For weak selection ($\gamma \ll 1$), we use the approximation $p^+(\gamma B) - p^+(\gamma) \approx \frac{1}{2}\gamma(1-B)$. Furthermore, note that $p^+(\gamma) \approx 1/2$ and $p^-(\gamma) \approx 1/2$, so $4p^+(\gamma)p^-(\gamma) \approx 1$. Thus, we can write:

$$\begin{aligned}\frac{W(\bar{G}_B)}{W(\bar{G})} &\approx 1 - \frac{L\gamma}{N}\frac{\gamma(1-B)}{2} && \text{[C.21]}\\ &= 1 - 4Ls\gamma(1-B)\left(\frac{1}{4}\right) && \text{[C.22]}\\ &\approx 1 - 4Ls\gamma(1-B)p^+(\gamma)p^-(\gamma). && \text{[C.23]}\end{aligned}$$

This result shows that for weakly selected sites, background selection drives a reduction in mean fitness proportional to the strength of selection, the total mutation rate, and the reduction in effective population size.

D. Changes in disease prevalence in the liability threshold model due to BGS

Derivation. Let V_E environmental variance, and let V_G be the genetic variance in the absence of background selection. The heritability in the absence of background selection is then

$$h^2 = \frac{V_G}{V_G + V_E}, \tag{D.1}$$

and the standardized threshold density is

$$\phi(T^*) = f(T)\sqrt{V_p}, \tag{D.2}$$

where $V_P = V_G + V_E = \frac{V_G}{h^2}$ is the total variance.

In the presence of background selection, the genetic variance is reduced to $V_{G,B} = BV_G$, and the phenotypic variance to $V_{P,B} = V_{G,B} + V_E$, so the heritability becomes

$$h_B^2 = \frac{V_{G,B}}{V_{P,B}} = \frac{V_{G,B}}{V_{G,B} + V_E} = \frac{BV_G}{BV_G + V_E}, \tag{D.3}$$

and we can write the standardized threshold density in the presence of background selection in terms of original quantities in its absence as

$$\phi(T_B^*) = f_B(T)\sqrt{V_{P,B}} = \frac{f(T)}{B}\sqrt{BV_G + V_E}, \tag{D.4}$$

where $T_B^* = \frac{T - \bar{G}_B}{\sqrt{V_{P,B}}}$.

Now, we can eliminate the V_E by solving Eq. (D.1) for $V_E = \frac{1-h^2}{h^2}V_G$ and substituting to get

$$\phi(T_B^*) = f_B(T)\sqrt{V_{P,B}} = \frac{f(T)}{B}\sqrt{V_G\left(\frac{1}{h^2} - (1-B)\right)}, \tag{D.5}$$

which is now written entirely in terms of counterfactual quantities from the equilibrium in the absence of background selection, and the B value. The increase in the standardized threshold density due to background selection is thus

$$\frac{\phi(T_B^*)}{\phi(T^*)} = \frac{\sqrt{1 - h^2(1-B)}}{B}, \tag{D.6}$$

Translating this into a statement about the effect on the prevalence requires that we make some assumption about the relationship between the standardized threshold density and the prevalence. The standard assumption is that the distribution of liability is Normal, in which case the two expressions for the standardized threshold density above can be plugged into

standard normal expressions to obtain the prevalence in each case. However, (9) argued that when the prevalence is low, the relationship between the standardized threshold density and the prevalence is very roughly linear. It follows that

$$\frac{\bar{R}_B}{\bar{R}} \approx \frac{\sqrt{1-h^2(1-B)}}{B}, \quad \text{[D.7]}$$

where $\bar{R}$ is the prevalence without BGS and $\bar{R}_B$ the prevalence with BGS (i.e. Eq. (17) in the main text). (9) also showed that if large effect sites make a substantial contribution to genetic variance in liability, the liability distribution may become skewed. However, due to the polygenic background, the far tail of the distribution will still be roughly Gaussian in shape, and it is the rapid Gaussian decay of the tail that is responsible for the fact that the change in threshold density roughly predicts the change in prevalence (because most of the density in the tail that is beyond the threshold is nonetheless very close to it), and so we expect Eq. (D.7) to hold, at least approximately, even if there is some skew in the distribution of liability.

Negligible impact of frequency spectrum skew. We also used simulations to explore whether this skew in the frequency spectrum impacts the disease prevalence. To do this, we varied the selection coefficients at the background selection sites while holding the mutation and recombination rates (and therefore the local effective size reduction) constant. We found that while the reduction in heterozygosity remained constant with increasing selection coefficients, the mean derived allele frequency among rare variants ($AF < 0.1$) rose, as expected. However, this increase in rare allele frequency had no discernible impact on the prevalence (Figure S4).

E. Solving the Two Effect-size Model with Background Selection

Here, we detail the method for solving the two-effect-size liability threshold model in the presence of background selection. (9) describes the method for solving this model in the absence of background selection in their Supplementary Section S7. Here, for completeness, we largely reproduced that description, with the addition of the BGS effect (as well as a few minor differences in notation).

In our two-effect model, we assume that a fraction $p_S = 1 - p_L$ of sites have small effects, while the remaining fraction p_L have large effects. We model the distribution of liability in the population as a convolution of two distributions. The first is a Normal component with variance $V_{G,B,S} + V_E$, where $V_{G,B,S}$ is the variance from small effect sites (in the presence of background selection), and V_E is the environmental variance. The second is a Poisson distribution on the number of large effect alleles that an individual carries. This Poisson distribution has mean

$$\lambda_L = \frac{2Lp_L\mu}{\delta(a_L)C}, \quad \text{[E.1]}$$

where

$$\delta(a_L) = F(T^* - a_L) - F(T^*) \quad \text{[E.2]}$$

is the risk effect of the large effect sites, $T^* = T - \bar{Z}$ is the distance between the mean and the threshold,

$$F(Z) = \sum_i P(i|\lambda_L) Q(Z|a_L(i-\lambda_L), V_{G,B,S} + V_E) \quad \text{[E.3]}$$

is the probability that an individual's liability exceeds a value of Z, where $Q(Z|u, \sigma^2) = 1 - \Phi(Z|u, \sigma^2)$ is the complementary CDF of a Normal distribution with mean u and variance σ^2, and $P(i|\lambda_L)$ is the probability that a Poisson random variable with mean λ_L takes a value of i. The density on total liability, in turn, is

$$f(Z) = \sum_i P(i|\lambda_L) \phi(Z|a_L(i-\lambda_L), V_{G,B,S} + V_E) \quad \text{[E.4]}$$

where $\phi(Z|u, \sigma^2)$ is the Normal PDF.

We solve two-effect model in the following way. First, we can solve for the fraction of sites fixed for the liability increasing allele at small effect sites. Because all large effect sites are fixed for the liability decreasing allele, this fraction is equal to

$$p^+(\gamma(a_S)) = \frac{\bar{a}p_T}{a_S p_S} \quad \text{[E.5]}$$

where

$$\bar{a} = a_S p_S + a_L p_L \quad \text{[E.6]}$$

is the mean effect size. The scaled selection coefficient of the small effect sites is determined entirely by the threshold induced fixation asymmetry and the resulting long-term fixation dynamics, and is therefore given by

$$\gamma(a_S) = \frac{1}{2} \ln \frac{1 - p^+(\gamma(a_S))}{p^+(\gamma(a_S))}, \quad \text{[E.7]}$$

and the threshold density by

$$f_B(T) = \frac{\gamma(a_S)}{2NBCa_S} = \frac{1}{4NBCa_S} \ln \frac{1-p^+(\gamma(a_S))}{p^+(\gamma(a_S))}. \qquad \text{[E.8]}$$

The genetic variance due to small effect sites is

$$V_{G,B,S} = \frac{\theta_L p_S B a_S b(\gamma(a_S))}{\gamma(a_S)}, \qquad \text{[E.9]}$$

where $b(\gamma(a_S)) = 1 - 2p^+(\gamma(a_S))$ is the degree of mutational asymmetry at small effect sites, $\theta_L = 4NL\mu$ is the total population scaled mutation rate of the trait.

We can then solve for the value of $\delta(a_L)$ via a line search. First, we plug T^* into the right hand side of Eq. (E.4) and set it equal to the right hand side of Eq. (E.8), yielding

$$\frac{1}{4NBCa_S} \ln \frac{1-p^+(\gamma(a_S))}{p^+(\gamma(a_S))} = \sum_i P(i|\lambda_L)\phi(T^*|a_L(i-\lambda_L), V_{G,B,S} + V_E). \qquad \text{[E.10]}$$

Then, for a proposed value of $\delta(a_L)$, we compute λ_L using Eq. (E.1), plug it into the RHS of Eq. (E.10), and numerically solve for the value of T^* that satisfies Eq. (E.10). We can then conduct a line search for the value of $\delta(a_L)$ that satisfies Eq. (E.2), given the value of T^* obtained from solving Eq. (E.10).

To constrain the liability-scaled heritability to a specific value, we add one additional step. Given the proposed value of $\delta(a_L)$, we compute the large effect contribution to the genetic variance as

$$V_{G,B,L} = a_L^2 \lambda_L \qquad \text{[E.11]}$$

and then compute the environmental variance as

$$V_E = \frac{1-h^2}{h^2}. \qquad \text{[E.12]}$$

Note that the inclusion of the B value in Eq. (E.8) and Eq. (E.9) to account for the impact of BGS on weakly selected sites represents the only substantial difference between the algorithm presented in (9) and the one presented here. The solution in the case of no BGS is therefore obtained simply by setting $B = 1$. To compare cases with and without background selection, we first solve the above system of equations for a fixed value of h^2 with $B = 1$, to obtain the solution in the absence of background selection. We then solve in the presence of background selection, holding the environmental variance (as opposed to the heritability), constant at the value we obtained in the no BGS case.

F. An Incidental Finding That the Bulmer Effect Increases the Genetic Variance in the Long Term

In our multi-locus stabilizing selection simulations, when we used the same recombination rate between causal loci as used in the simulation of the liability threshold model (5×10^{-6}), the genetic diversity reduction of causal sites under background selection initially did not match our expectations based on our theory and our single-locus simulations (Figure S11). We hypothesized that this might be related to the Bulmer effect (3, 10), i.e. the accumulation of negative linkage disequilibrium between causal sites due to selection. More precisely, the genetic variance can be composed into two components:

$$Var\left(\sum_j a_j g_j\right) = \sum_j a_j^2 Var(g_j) + \sum_j \sum_{k \neq j} a_j a_k Cov(g_j, g_k), \qquad \text{[F.1]}$$

where the first term is the additive genic variance and the second term captures the contribution of linkage disequilibrium among causal sites. Under neutrality, the expectation of the LD term is equal to zero. However, because stabilizing selection acts most strongly against individuals carrying combinations of alleles that result in extreme phenotypes, it causes negative covariance among alleles of like sign, leading to a negative expectation for the contribution of the LD term. Thus, while stabilizing selection is acting, it leads to a reduction in the total genetic variance due to this negative LD. The reduction in genetic variance due to this negative LD is what is known as the “Bulmer effect”. Because recombination will quickly eliminate this negative LD if stabilizing selection ceases, the Bulmer effect is generally understood as a “short term” phenomenon.

For any given focal allele, the negative LD induced by the Bulmer effect leads to an attenuation of the allele’s marginal correlation with the phenotype, relative to its causal effect (3, 6). In the modern statistical genetics literature, this effect is sometimes known as “linkage masking” (11). Because the expected change in the frequency of an allele from a given generation to the next depends on its marginal correlation with fitness in that generation, rather than its causal effect on fitness, the attenuation of the marginal phenotypic correlation in turn leads to an attenuation of the selection coefficient, and this in turn leads to a reduction in the magnitude of the expected change in frequency due to selection. More precisely, whereas in the absence of any Bulmer effect, the expected change in the minor allele frequency is given by:

$$\mathbb{E}(\Delta x) \approx -t(a)x(1-x)(1-2x), \qquad \text{[F.2]}$$

with $t(a) = \frac{a^2}{2\omega^2}$, in its presence we expect that $t(a) < \frac{a^2}{2\omega^2}$, where the magnitude of the reduction in $t(a)$ depends on the amount of negative LD. This slowdown in the pace of frequency change is studied in greater depth by (5).

This effect has an intriguing implication. That is, while the negative LD induced by stabilizing selection causes a short-term reduction in the genetic variance relative to the genic variance, over long timescales, we would expect it to increase in the genic variance, due to the weaker selection on individual variants. Whether the net effect is to increase or decrease the genetic variance then depends on which effect is larger.

We hypothesized that this phenomenon may be responsible for the difference that we initially observed between our predictions and our simulation results. To investigate this hypothesis, we performed simulations under the stabilizing selection model without background selection and varied the rate of recombination between the neighboring causal sites (Figure S11). When the recombination rate is low, we find that the genetic variance is reduced relative to the genic variance, but is nevertheless increased relative to what we observed with higher recombination rates (which match the theoretical predictions based on unlinked theory; (12)). Thus, our simulation results suggest that over long time scales, the net impact of the Bulmer effect is to increase the genetic variance, as the increase in the genic variance caused by the slowdown in the pace of allele frequency change is larger than the reduction in variance due to the negative LD. Moreover, when we increased the recombination rate in our study of background selection to a level at which there was no longer any Bulmer effect or inflation of the genic variance, we found that our theoretical prediction for the impact of background selection then matched our theoretical predictions (Figure 5A), indicating that the Bulmer effect was indeed the culprit.

Our primary focus was on understanding the impact of background selection, so we did not study this effect any further. However, it is worth noting that, in addition to (5), at least two prior publications have also studied this effect. Specifically, (13) found that in his Gaussian allelic model, the decrease in genetic variance due to the Bulmer effect is exactly offset by the increase in genic variance, leading to no net effect. In contrast, (14), employing their "rare alleles" approximation, do predict an increase in the genetic variance when recombination rates are low, but their predictions do not match our simulation results. A more complete analysis of this phenomenon would be valuable.

G. Variation in a and B in the Threshold Model

In this section, we provide more explicit mathematical support for the arguments in the main text for the cases with variation in local B value. Here, we start from a slightly more general position than in the main text, by assuming that the effects and the local N_e reductions have some joint distribution $g(a, B)$. Then analogous to Eq. (18) in the main text, equilibrium is established by evolving the value of $f(T)$ that solves

$$p_T = \int\int g(a,B)\frac{a}{\bar{a}}p^+\left(2NC \cdot f(T) \cdot aB\right)\mathrm{d}a\mathrm{d}B. \quad \text{[G.1]}$$

Effectively neutral regime. When $p_T \approx \frac{1}{2}$, the fixation asymmetry is approximately linear, $p^+(\gamma) \approx \frac{1}{2}(1-\gamma)$. Inserting this approximation and evaluating the resulting integral, we have

$$p_T \approx \frac{1}{2} - \frac{NCf_B(T)}{\bar{a}}\mathbb{E}[a^2B], \quad \text{[G.2]}$$

which we can solve for

$$f_B(T) \approx \left(\frac{\frac{1}{2}-p_T}{NC}\right)\left(\frac{\bar{a}}{\mathbb{E}[a^2B]}\right). \quad \text{[G.3]}$$

In the absence of background selection, $B = 1$, so

$$f(T) \approx \left(\frac{\frac{1}{2}-p_T}{NC}\right)\left(\frac{\bar{a}}{\mathbb{E}[a^2]}\right). \quad \text{[G.4]}$$

The global compensation factor is

$$\frac{f_B(T)}{f(T)} \approx \frac{\mathbb{E}[a^2]}{\mathbb{E}[a^2B]}, \quad \text{[G.5]}$$

and is thus an average of B that is weighted by the squared effect sizes, reflecting the fact that variance contributions scale with a^2 in the neutral regime. If a and B are independent, then

$$\frac{f_B(T)}{f(T)} \approx \frac{\mathbb{E}[a^2]}{\mathbb{E}[a^2]\mathbb{E}[B]} = \frac{1}{\mathbb{E}[B]}. \quad \text{[G.6]}$$

Strong selection limit. In the strong selection limit, the behavior depends on whether the coefficient of variation of the effect distribution is large or small. Let us first consider the small coefficient of variation case.

$g_a(a)$ ***has a small coefficient of variation.*** In this case, the distribution of scaled selection coefficients γ is centered over a relatively narrow range. Most sites will have similar scaled selection coefficients, and the bulk of their distribution moves from the weak into the strong selection regime as $p_T \to 0$. In this regime, $p^+(\gamma) = \left(1 + e^{2\gamma}\right)^{-1} \approx e^{-2\gamma}$, so Eq. (G.1) becomes

$$p_T = \frac{1}{\bar{a}} \mathbb{E}\left[ae^{-4NCf(T)\cdot aB}\right]. \tag{G.7}$$

The exponential decay dominates over the linear factor of a, and so the primary contribution to the expectation is from sites with the minimum value of aB, i.e. the sites with the smallest effect sizes experiencing the strongest background selection. This implies that

$$p_T \approx K_1 e^{-4NCf(T)\cdot(aB)_{min}} \tag{G.8}$$

where K_1 is a constant, and $(aB)_{min}$ is the minimum of the product of aB across sites. Solving for $f(T)$, we have

$$f_B(T) \approx \frac{\ln\left(\frac{p_T}{K_1}\right)}{4NC} \frac{1}{(aB)_{min}} \tag{G.9}$$

and

$$f(T) \approx \frac{\ln\left(\frac{p_T}{K_1}\right)}{4NC} \frac{1}{a_{min}}, \tag{G.10}$$

so that the global compensation factor is

$$\frac{f_B(T)}{f(T)} \approx \frac{a_{min}}{(aB)_{min}}. \tag{G.11}$$

If a and B are independent, then $(aB)_{min} = a_{min}B_{min}$, and so

$$\frac{f_B(T)}{f(T)} \approx \frac{1}{B_{min}}. \tag{G.12}$$

$g_a(a)$ ***has a large CV.*** Alternatively, if there is high variance in the effect distribution, then the scaled selection coefficients of sites will not all cluster together in the same selection regime, but rather will be spread across all of the selection regimes, with a large subset of small effect sites belonging to the effectively neutral regime, even as $p_T \to 0$. As a result, we must consider contributions from the whole distribution, rather than just a narrow slice of it. Here, it is not clear *a priori* how to obtain any general results for an arbitrary joint distribution, $g(a, B)$, so we will assume they are independent (i.e., $g(a, B) = g_a(a)g_B(B)$). As a tractable choice for a distribution which can model the high variance scenario of interest, suppose that the effects follow a gamma distribution with shape and scale parameters k and ω, i.e. $g_a(a) = \frac{1}{\Gamma(k)\omega^k} a^{k-1} e^{-\frac{a}{\omega}}$. The coefficient of variation of the gamma distribution is equal to $k^{-1/2}$, so we will be interested in the $k \to 0$ limit, though we proceed generally for now, and consider this limit below.

Although the entire distribution contributes to the integral, the exponential suppression of the transition into the strongly selected regime still dominates, so we can still make the same $p^+(\gamma) \approx e^{-2\gamma}$ approximation that we made in the low CV case. Eq. (G.1) becomes

$$p_T\bar{a} = \frac{1}{\Gamma(k)\omega^k} \int_B g_B(B) \left[\int_a a^k e^{-4NCf_B(T)aB - \frac{a}{\omega}} da\right] dB. \tag{G.13}$$

Because we are interested in the strong selection limit (i.e. $p_T \to 0$), we assume that the threshold density $f_B(T)$ is large enough that $4NCf_B(T)B \gg \frac{1}{\omega}$. In physical terms, this inequality implies that the fixation asymmetry $p^+(\gamma)$ decays with increasing effect size a much more rapidly than the mutational input $g_a(a)$ does. That is, selection effectively prevents the fixation of deleterious alleles with moderate effect sizes well before such alleles become rare in the mutational distribution. Consequently, the integral is dominated by the interaction between selection and the power-law portion of the effect size distribution, allowing us to ignore the exponential suppression of the tail of the gamma distribution. Mathematically, this allows us to approximate the exponential function in Eq. (G.13): $e^{-4NCf_B(T)aB - \frac{a}{\omega}} \approx e^{-4NCf_B(T)aB}$, so that we can write

$$p_T\bar{a} = \frac{1}{\Gamma(k)\omega^k} \int_B g_B(B) \left[\int_a a^k e^{-4NCf_B(T)aB} da\right] dB. \tag{G.14}$$

We can then use u substitution (set $u = 4NCf_B(T)aB$ so that $a = \frac{u}{4NCf_B(T)B}$ and $da = \frac{du}{4NCf_B(T)B}$) to rewrite the inner integral as

$$\int_a a^k e^{-4NCf(T)aB} da \approx \left[\frac{1}{(4NCf_B(T)B)^{k+1}}\right] \int_0^\infty u^k e^{-u} du, \tag{G.15}$$

where the integral in u is the gamma function, i.e. $\int_0^\infty u^k e^{-u} du = \Gamma(k+1)$. We can therefore write Eq. (G.14) as

$$p_T \bar{a} \approx \frac{k}{\omega^k [4NCf_B(T)]^{k+1}} \int_B g_B(B) \left[\frac{1}{B^{k+1}}\right] dB, \qquad \text{[G.16]}$$

which we can solve to find

$$f_B(T) \approx \left[\frac{k^{\frac{1}{k+1}}}{4NC(\omega^k p_T \bar{a})^{\frac{1}{k+1}}}\right] \mathbb{E}\left[B^{-(k+1)}\right]^{\frac{1}{k+1}} \qquad \text{[G.17]}$$

implying that

$$\frac{f_B(T)}{f(T)} \approx \mathbb{E}\left[B^{-(k+1)}\right]^{\frac{1}{k+1}}. \qquad \text{[G.18]}$$

Now, let us consider the large coefficient of variation limit. As $k \to 0$, the gamma distribution follows a power law that decays at rate a^{-1} until cutoff by the exponential suppress term $e^{-\frac{a}{\omega}}$. In this case, the effect size distribution is very broad, and $\frac{f_B(T)}{f(T)}$ is equal to the expectation of B^{-1}:

$$\frac{f_B(T)}{f(T)} \approx \mathbb{E}\left[B^{-1}\right]. \qquad \text{[G.19]}$$

Alternatively, as $k \to \infty$, the variance of the effect size distribution shrinks, and $\frac{f_B(T)}{f(T)}$ converges toward the minimum,

$$\frac{f_B(T)}{f(T)} \approx \frac{1}{B_{min}} \qquad \text{[G.20]}$$

consistent with our previous results.

Finally, we must address the validity of the continuous approximation in the high-CV limit. In our continuous mathematical model, the Gamma distribution provides an effectively infinite number of sites with vanishingly small effect sizes. Consequently, the compensation process never terminates because there are always sites small enough to remain in the effectively neutral regime. In a physical genome with a finite number of sites L, there is necessarily a single site with the minimum effect size, a_{min}. Technically, the compensation process described above would terminate once $f(T)$ becomes large enough that even this smallest effect is pushed into the strong selection regime. However, this termination occurs only at the limit where selection is so strong that carrying a single allele at any site with any effect size is sufficient to cross the threshold (i.e., $s \approx C$). Thus, the limit where this global compensation ultimately stops acting is precisely the limit where the epistasis becomes unimportant, and the model collapses onto one where all loci impact fitness independently in a Mendelian fashion. Thus, by invoking a model with threshold epistasis and wide variation in effect sizes in the context of a finite genome, we are effectively invoking the $p_T \to 0$ limit, but assuming that p_T nonetheless remains large enough that $a_{min}/2L\bar{a} \ll p_T$. As shown in Figure 4A, explicit numerical solutions of Eq. (G.1) exhibit convergence to the $f_B(T)/f(T) \approx \mathbb{E}\left[B^{-1}\right]$ limit at relatively large values of p_T once the CV becomes moderately large, so this assumption is entirely appropriate.

Impact on the genetic variance. Having determined the global compensation factor, we can now derive the impact of background selection on the genetic variance contributed by specific sites. Let $B_{global} = \frac{f(T)}{f_B(T)}$ be the effective genome-wide reduction in the threshold density (note that this is the inverse of the compensation ratio derived above, e.g., $B_{global} = \frac{1}{\mathbb{E}[B^{-1}]}$ in the high-CV gamma limit).

Consider a specific site i with effect size a and a local effective population size reduction B_{local}. In the absence of background selection, the site has a scaled selection coefficient $\gamma = 2Naf(T)C$. In the presence of background selection, the unscaled selection coefficient increases due to the global shift in threshold density:

$$s_B \approx af_B(T)C = af(T)\frac{1}{B_{global}}C = \frac{s}{B_{global}}. \qquad \text{[G.21]}$$

However, the local effective population size decreases: $N \to NB_{local}$. The new scaled selection coefficient is therefore:

$$\gamma_B = 2(NB_{local})s_B = 2NB_{local}\frac{s}{B_{global}} = \gamma\frac{B_{local}}{B_{global}}. \qquad \text{[G.22]}$$

This ratio $\frac{B_{local}}{B_{global}}$ determines whether the site experiences a net increase or decrease in the efficiency of selection relative to drift.

The genetic variance contributed by this site is proportional to the mutation rate and the function $b(\gamma)/\gamma$, where $b(\gamma) = \tanh(\gamma)$. Accounting for the reduction in the effective mutation rate ($\theta \to \theta B_{local}$), the variance in the presence of background selection is:

$$\begin{aligned} V_{G,B} &= \frac{\theta B_{local}}{\gamma_B} b(\gamma_B) \\ &= \frac{\theta B_{local}}{\gamma \frac{B_{local}}{B_{global}}} b\left(\gamma \frac{B_{local}}{B_{global}}\right) \\ &= B_{global} \frac{\theta}{\gamma} b\left(\gamma \frac{B_{local}}{B_{global}}\right). \end{aligned} \tag{G.23}$$

Comparing this to the original variance $V_G = \frac{\theta}{\gamma} b(\gamma)$, we obtain the reduction factor:

$$\frac{V_{G,B}}{V_G} = B_{global} \frac{b\left(\gamma \frac{B_{local}}{B_{global}}\right)}{b(\gamma)}, \tag{G.24}$$

i.e. Eq. (22) in the main text.

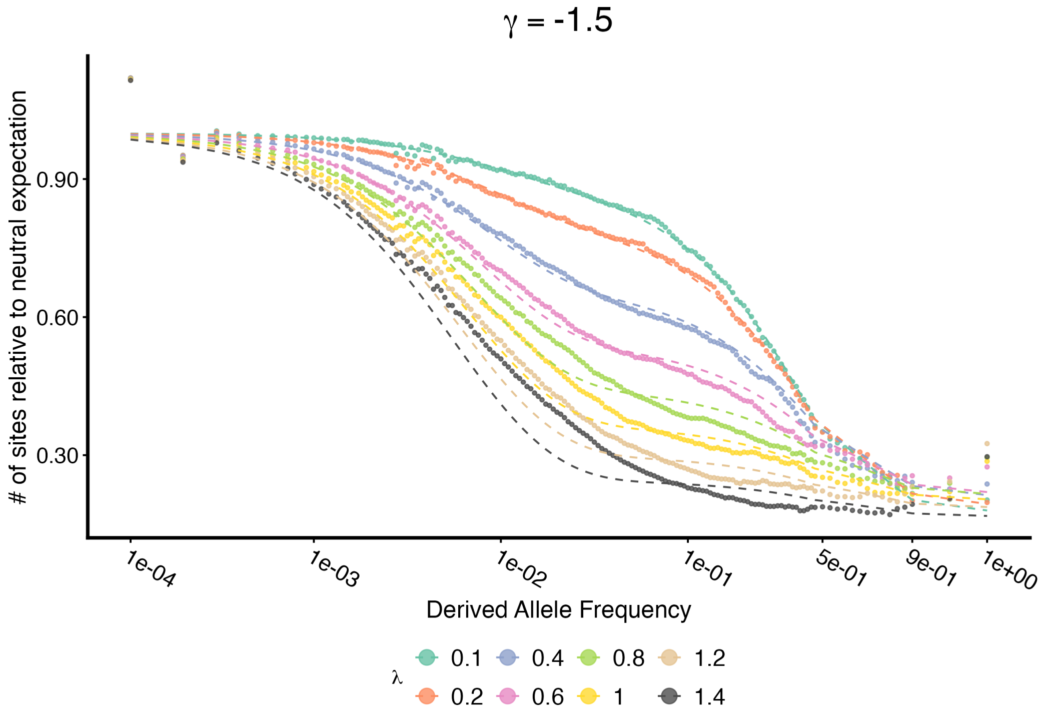

Fig. S1. Robustness of the frequency-dependent BGS approximation across varying mutation intensities (λ). The site frequency spectrum (SFS) of a focal site with $\gamma = -1.5$, scaled relative to the standard neutral expectation, is shown for simulations with varying values of the background mutation flux $\lambda = 2\nu/r$. Points represent simulation results, while solid lines show the analytical prediction using the $B(q)$ approximation derived in the weak mutation limit (Eq. (B.27)). Although the analytical model is formally derived for the limit where mutation is weak relative to recombination ($\lambda \ll 1$), it accurately captures the distortion of the SFS for $\lambda < 1/2$. Departures from the prediction for $\lambda > 1/2$ indicate the onset of the "fitness drag" regime driven by the accumulation of new mutations, which we describe in Supplementary Text B.

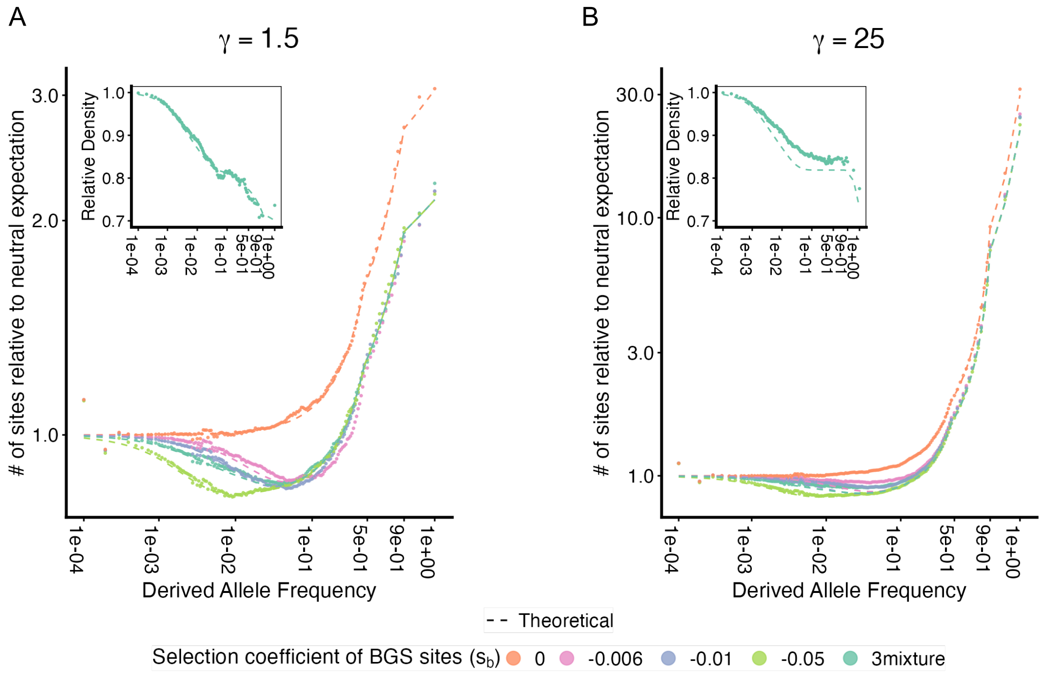

Fig. S2. The site frequency spectrum (SFS) for positively selected focal alleles, scaled to the neutral allele expectation, is shown for a BGS intensity of $B \approx 0.82$. Points represent simulation results, while dashed lines indicate theoretical predictions based on the frequency-dependent $B(q)$ approximation (Eq. (B.32)). We show results for three different selection coefficients of background selection sites ($s_b = -0.006, -0.01, -0.05$), as well as an equal mixture of all three. The insets show the relative density of alleles across frequency bins, comparing scenarios with BGS to those without. Focal selected alleles are under (A) weak positive selection ($\gamma = 1.5$) and (B) intermediate positive selection ($\gamma = 25$). When $\gamma = 25$, simulations with weaker BGS ($|s_b|$ < 0.05) exhibit a deviate from the theoretical line. This occurs because when the strength of direct selection on the focal allele is similar to the strength of background selection ($|\gamma| \approx |2Ns_b|$), the two processes act on similar timescales. This transits into the regime where the focal allele is dominated by its own fitness effect before the background is purged (Eq. (B.35)).

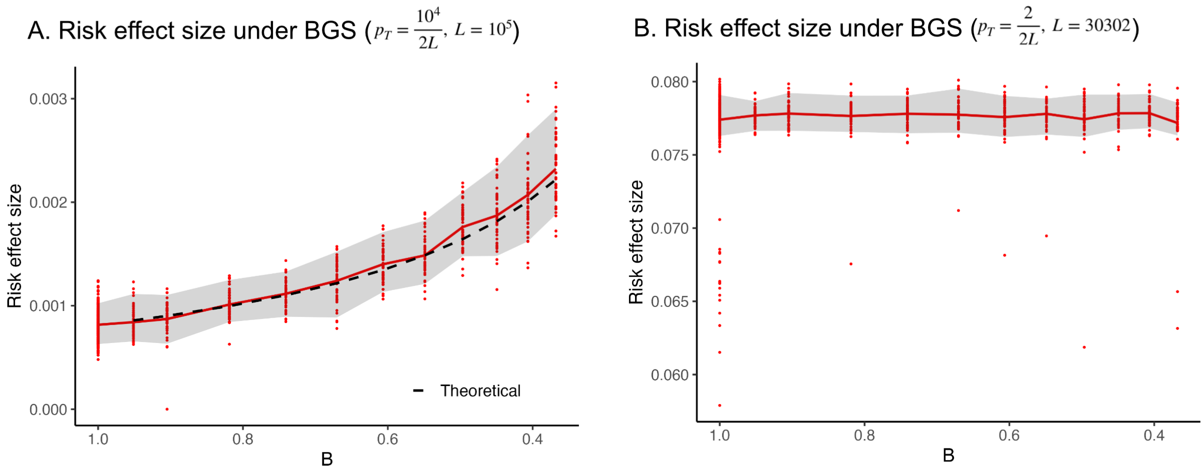

Fig. S3. The impact of BGS on risk effect size in the single-effect liability threshold model. (A) When $p_T \gg \frac{1}{2L}$, the risk effect size increases with increasing strength of BGS. The black dashed line shows the theoretical prediction of $f(T) \to f(T)/B$. (B) When $p_T \sim \frac{1}{2L}$, the risk effect size does not change under BGS.

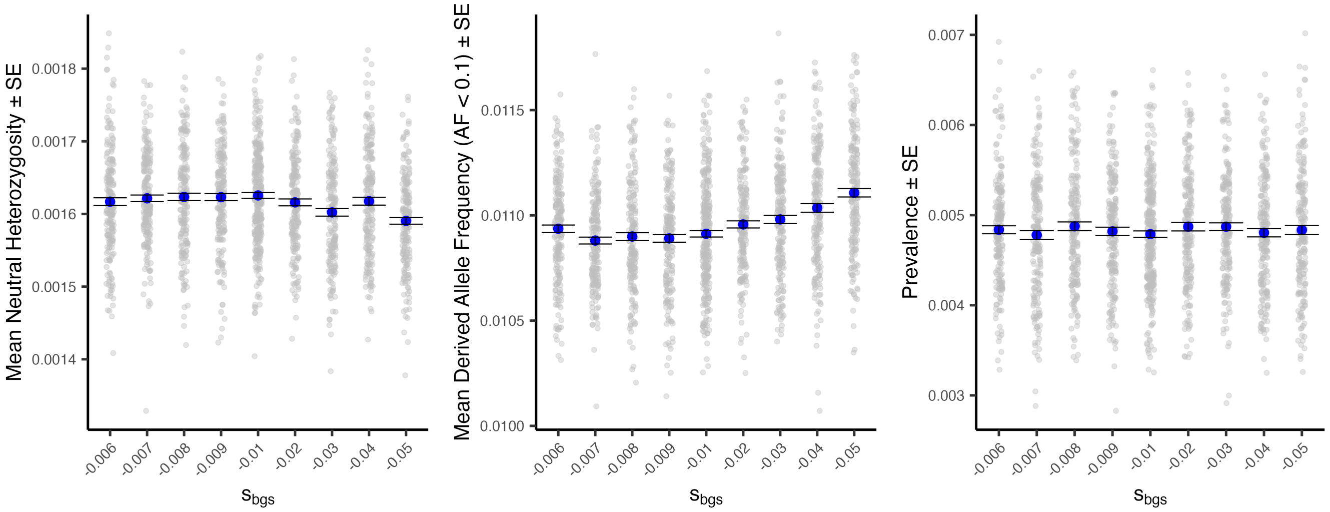

Fig. S4. Simulations were designed to isolate the effect of background selection–induced distortion of the site-frequency spectrum (SFS), particularly the excess of rare variants, on disease prevalence under the liability-threshold model. Mutation rate ($\nu = 2 \times 10^{-8}$) and recombination rate ($r = 2 \times 10^{-7}$) at BGS sites were held fixed, while only the selection coefficient (s_b) was varied. The left panel shows that mean neutral heterozygosity is insensitive to s_b. The center panel shows that weakening selection at BGS sites leads to a decrease in the mean derived-allele frequency among rare variants. Despite this shift in the SFS, the disease prevalence is not minimally affected, as shown in the right panel.

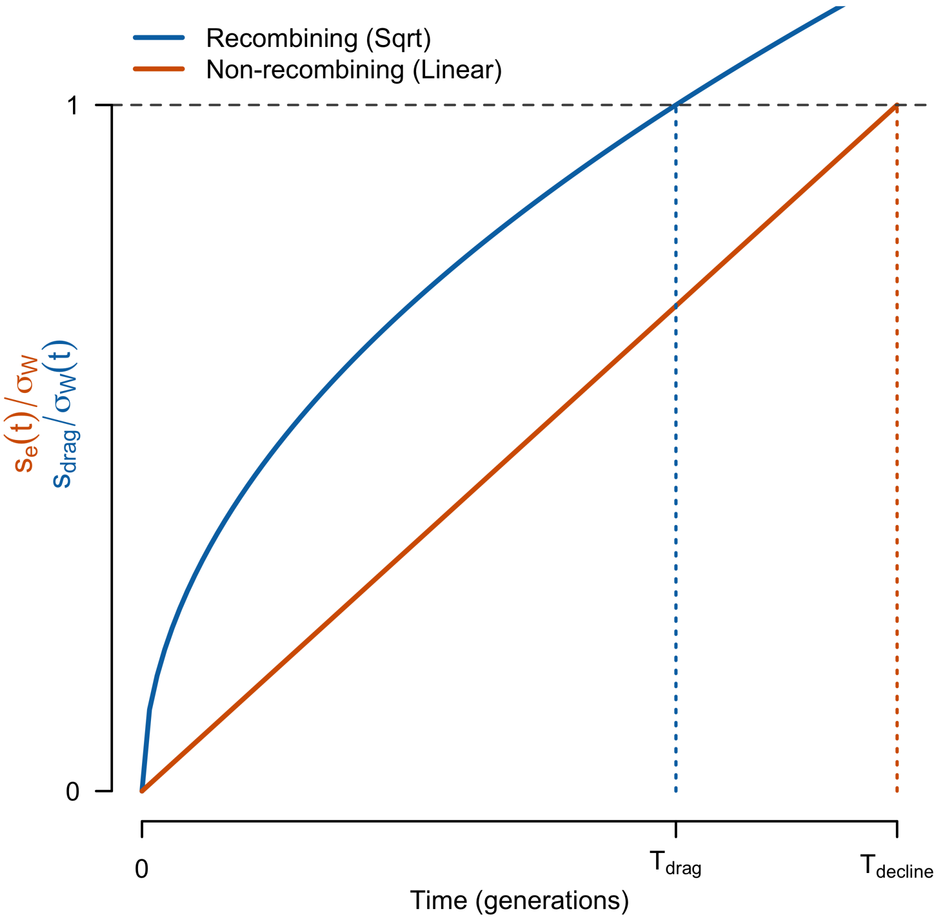

Fig. S5. Dynamics of the onset of effective selection in recombining vs. non-recombining models. This figure compares the time evolution of the ratio of the effective selection coefficient (s_{eff}) to the relevant standard deviation of fitness (σ_W) for a newly arisen allele. In the non-recombining model (orange), the fitness deficit accumulates linearly ($s_{eff}(t) \propto t$) against a constant background variation, leading to a linear increase in the ratio. The threshold for effective selection ($T_{decline}$) is reached when this ratio equals 1. In the recombining model (blue), the fitness deficit is constant ($s_{drag} = \lambda s_b$), but the relevant background variation shrinks as the associated haplotype block shortens ($\sigma_W(t) \propto t^{-1/2}$), causing the ratio to grow with $\sqrt{t}$. This dynamic leads to a more rapid onset of effective selection, both because $T_{drag} < T_{decline}$ and because the functional form of the ratio results in faster growth of the relative fitness deficit in early generations. Together, these factors imply that the frequency spectrum is distorted at significantly lower frequencies in recombining genomes. For the example plotted here, $\lambda = 2$.

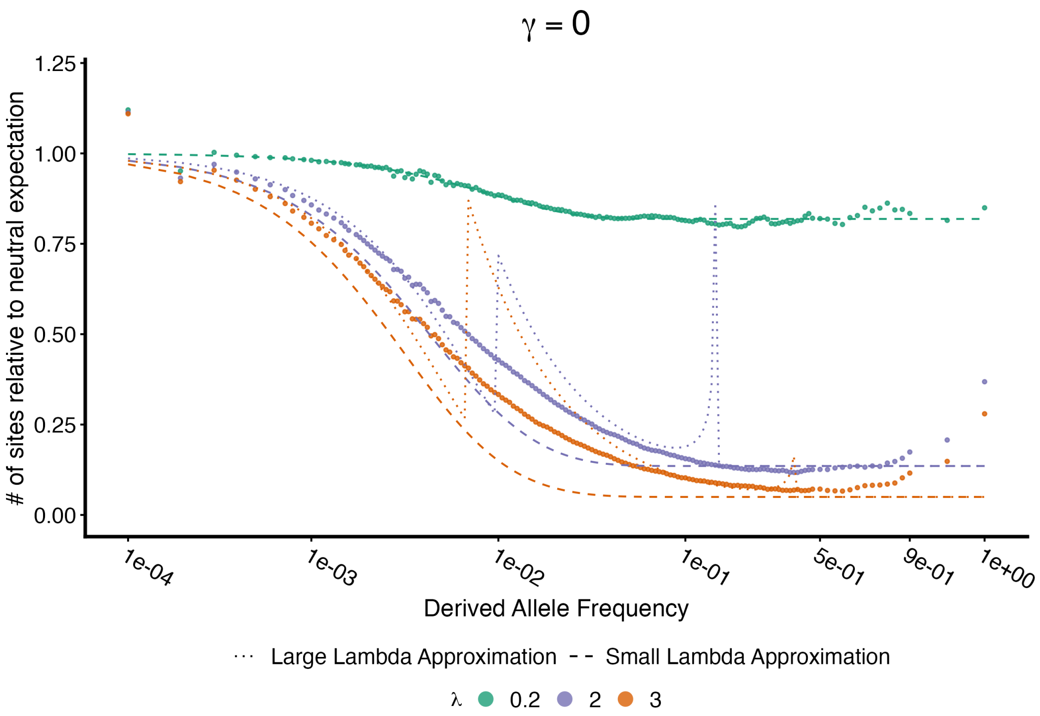

Fig. S6. The site frequency spectrum (SFS) of a neutral focal site ($\gamma = 0$) is shown relative to the standard neutral expectation for three different background selection intensities. Points represent simulation results for various levels of background mutation flux ($\lambda = 2\nu/r$), ranging from weak ($\lambda = 0.2$) to strong ($\lambda = 2, 3$). For low mutation flux ($\lambda = 0.2$), the distortion of the SFS is well-captured by the weak mutation/strong recombination (small lambda) approximation with $\mathrm{Eq.}$ $(\mathrm{B.24})$ (dashed lines), which accounts for selection acting primarily against deleterious alleles already present on the initial background. As the mutation flux increases ($\lambda > 1/2$), "fitness drag" regime kicks in and the dynamics are instead better described by the strong mutation/weak recombination (large lambda) approximation with ($\mathrm{Eq.}$ $(\mathrm{B.47})$)(dotted lines), which accounts for the continuous accumulation of fitness drag on the shortening associated haplotype block. Notably, the spikes in the large lambda approximation represent logarithmic divergences near the transitions between frequency regimes in $\mathrm{Eq.}$ $(\mathrm{B.47})$, where (7) applied a smoothing technique to resolve these boundaries.

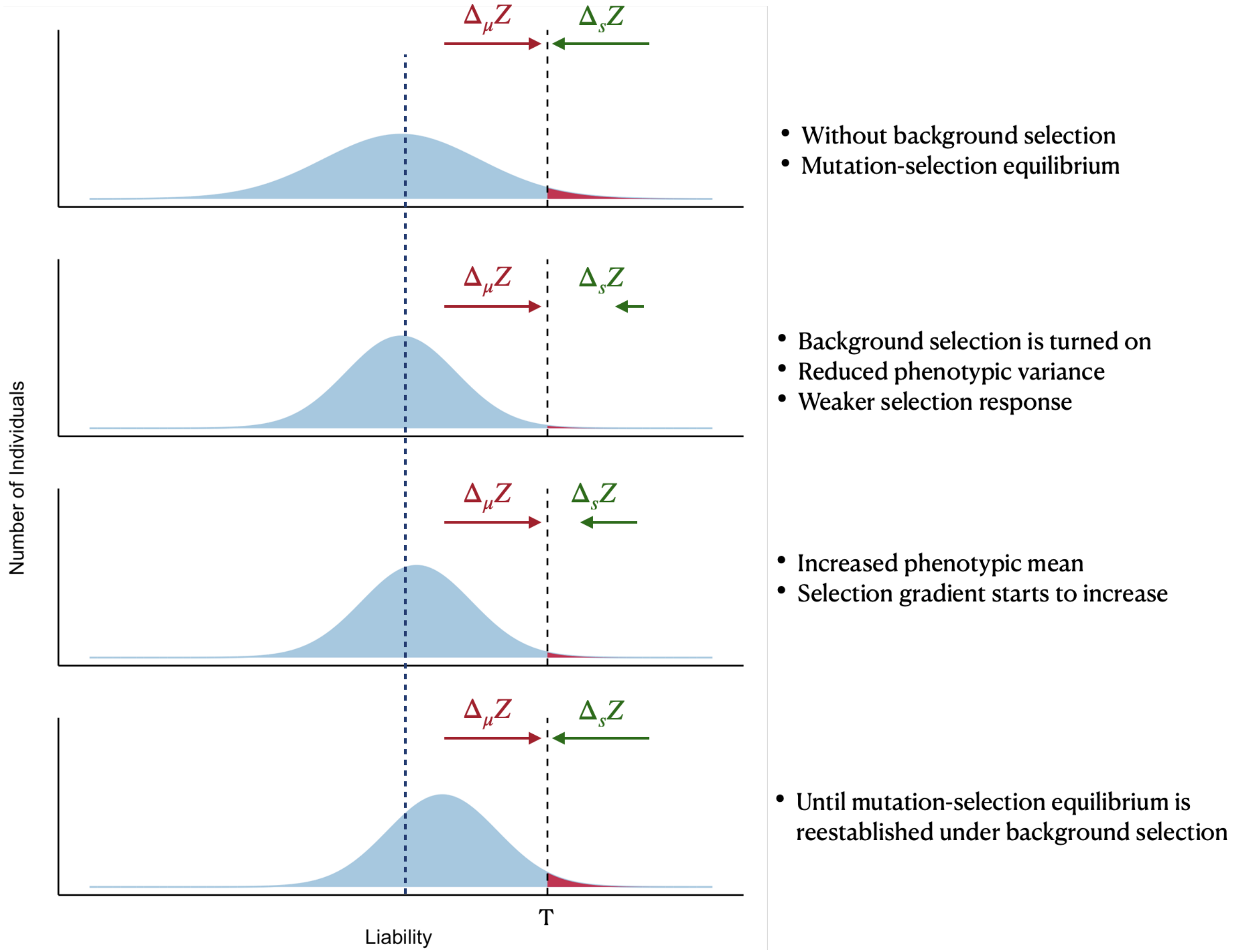

Fig. S7. A cartoon illustration that explains how background selection impacts disease prevalence. This illustration imagines a dynamic process where the population is first at equilibrium in the absence of background selection, before it is "turned on", and the population evolves to the new equilibrium. In reality, top and bottom panels should be viewed as counterfactual comparison in a model without vs with background selection.

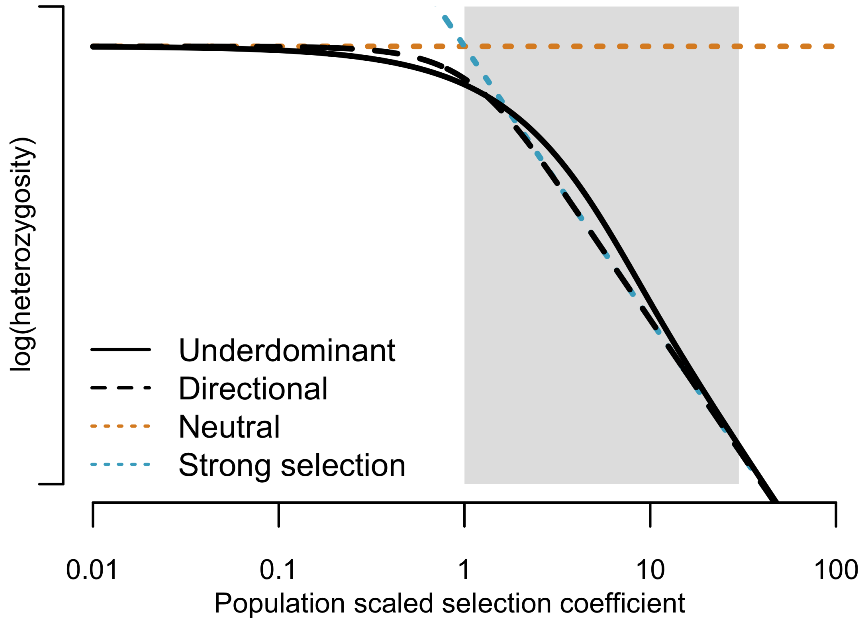

Fig. S8. A comparison of expected heterozygosity for alleles with directional selection (Eq. (6), dashed black line) vs. under dominant selection (Eq. (23), solid black line) at different scaled selection coefficients. The expected neutral heterozygosity is θ, and the expected heterozygosity for strong selection is $\frac{\theta}{\gamma}$.

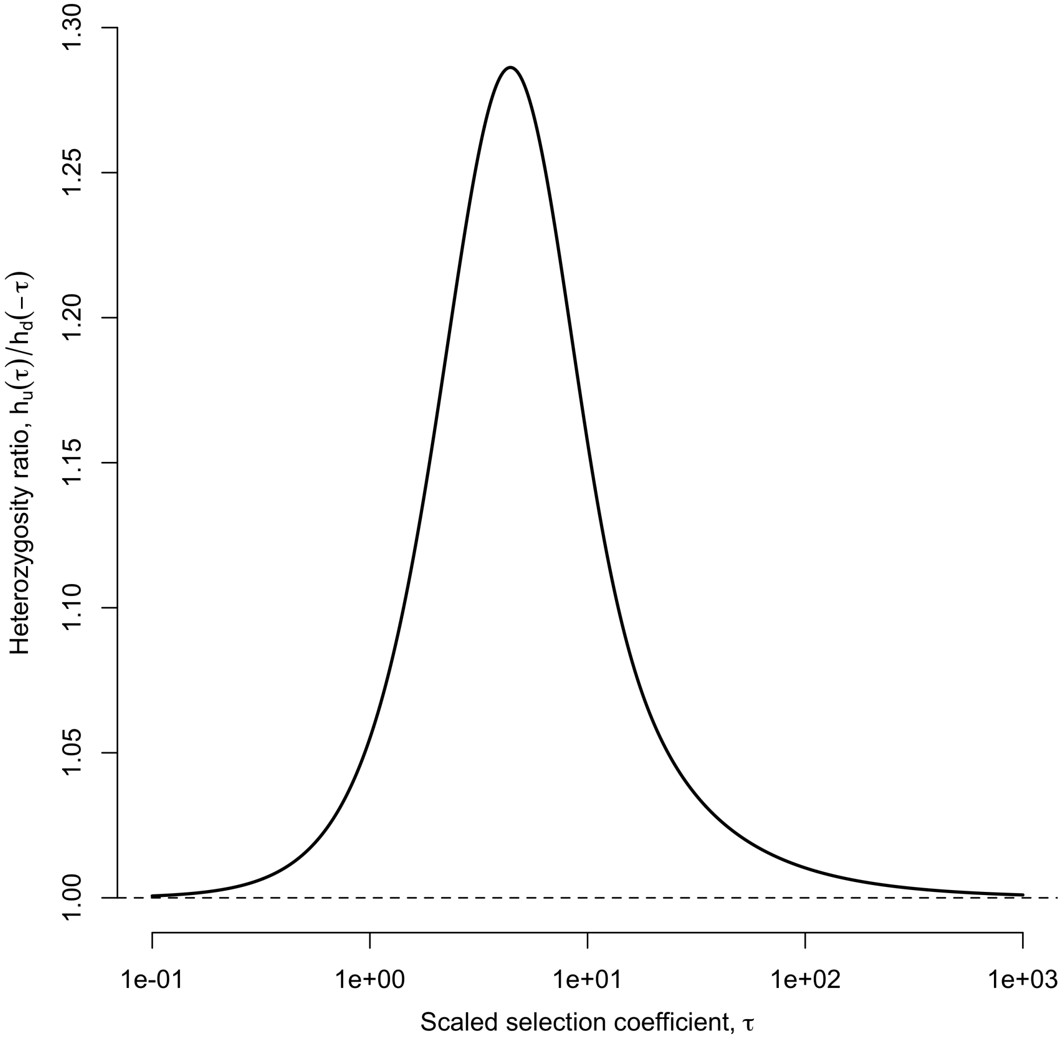

Fig. S9. The ratio $h_u(\tau)/h_d(-\tau)$, measuring the increase in heterozygosity due to the underdominance-induced slow down in allele frequency change, as a function of τ.

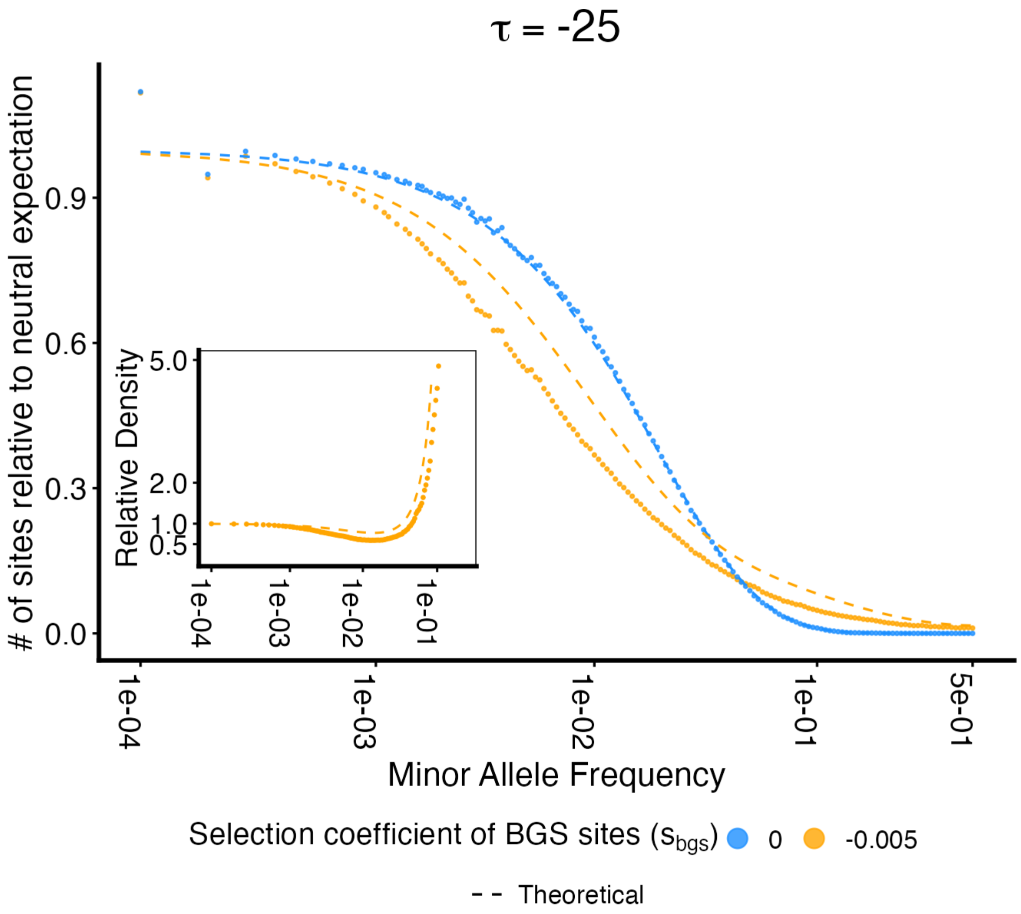

Fig. S10. Site frequency spectrum (upper panel) and relative density (lower panel) of a selected allele with under-dominant selection $2Nt = -25$, with a background selection intensity $B \approx 0.2$.The theoretical results (dashed line) obtained with frequency dependent B approximation using Eq. (B.26) breaks down in this large mutation/small recombination regime and does not predict the simulation well.

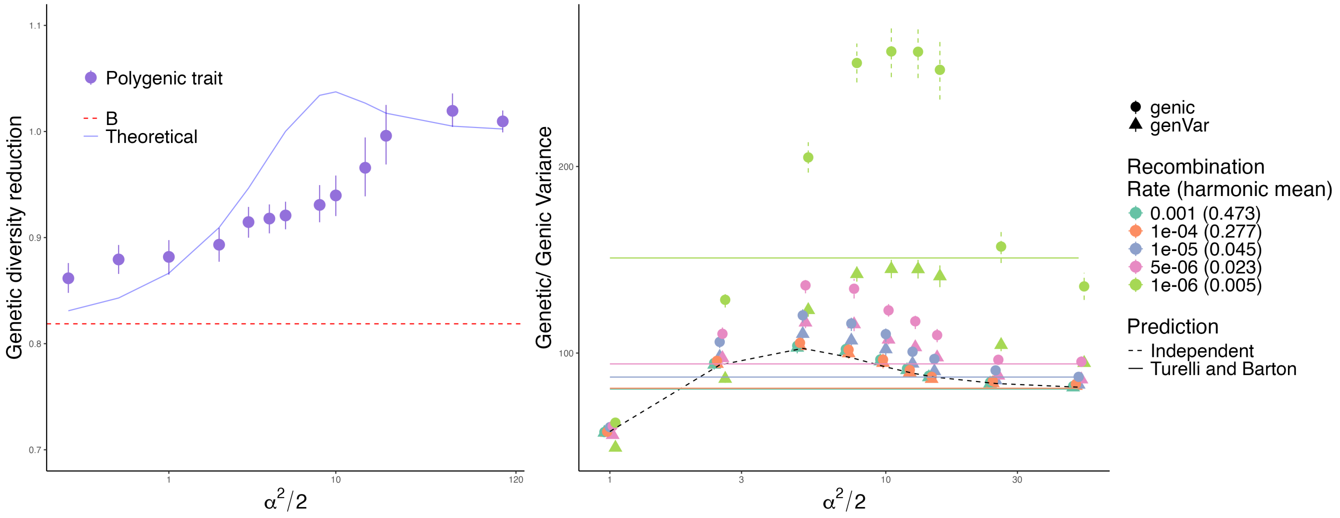

Fig. S11. Left panel: we simulated polygenic traits evolving under stabilizing selection and set the recombination rate between causal loci at 5×10^{-6} and mutation rate at 2×10^{-8}. With these parameters choices, we found that the effect of BGS on the genetic variance of the trait was different from the single-site prediction. Right panel: we simulated traits under stabilizing selection without background selection and varied the recombination rates between causal loci. The mutation rate is fixed at 2×10^{-8}. We plot the genic variance (circle) and genetic variance (triangle) and compare them to the predicted genetic variance that assumes independent causal loci (dashed line), and to the "rare alleles" approximation from (14) (solid lines). While the LD component of genetic variance is negative, as expected, due to the slow down mechanism described by (5) the genic variance is increased by more than the negative LD subtracts, so that the net effect on the genetic variance is to increase it.

References

1. BC Haller, PW Messer, Slim 3: forward genetic simulations beyond the wright–fisher model. *Mol. biology evolution* **36**, 632–637 (2019).
2. B Charlesworth, M Morgan, D Charlesworth, The effect of deleterious mutations on neutral molecular variation. *Genetics* **134**, 1289–1303 (1993).
3. M Bulmer, The effect of selection on genetic variability. *The Am. Nat.* **105**, 201–211 (1971).
4. M Bulmer, Linkage disequilibrium and genetic variability. *Genet. Res.* **23**, 281–289 (1974).
5. S Negm, C Veller, The effect of long-range linkage disequilibrium on allele-frequency dynamics under stabilizing selection. Preprint at bioRxiv https://www.biorxiv.org/content/10.1101/2024.06.27.601075v1 (2024).
6. C Veller, GM Coop, Interpreting population-and family-based genome-wide association studies in the presence of confounding. *PLoS Biol.* **22**, e3002511 (2024).
7. I Cvijović, BH Good, MM Desai, The effect of strong purifying selection on genetic diversity. *Genetics* **209**, 1235–1278 (2018).
8. B Charlesworth, The effect of background selection against deleterious mutations on weakly selected, linked variants. *Genet. Res.* **63**, 213–227 (1994).
9. JJ Berg, X Li, K Riall, LK Hayward, G Sella, Mutation–selection–drift balance models of complex diseases. *Genetics* **231**, iyaf220 (2025).
10. M Bulmer, The effect of selection on genetic variability: a simulation study. *Genet. Res.* **28**, 101–117 (1976).
11. BC Brown, AL Price, NA Patsopoulos, N Zaitlen, Local Joint Testing Improves Power and Identifies Hidden Heritability in Association Studies. *Genetics* **203**, 1105–1116 (2016).
12. YB Simons, K Bullaughey, RR Hudson, G Sella, A population genetic interpretation of gwas findings for human quantitative traits. *PLoS biology* **16**, e2002985 (2018).
13. R Lande, The maintenance of genetic variability by mutation in a polygenic character with linked loci. *Genet. Res.* **26**, 221–235 (1975).
14. M Turelli, NH Barton, Dynamics of polygenic characters under selection. *Theor. Popul. Biol.* **38**, 1–57 (1990).
